# Ex vivo modeling of precision immuno-oncology responses in lung cancer

**DOI:** 10.1126/sciadv.adq6830

**Published:** 2024-10-30

**Authors:** Bassel Alsaed, Johannes Smolander, Hanna Laitinen, Linh Lin, Nina Bobik, Lilja Lahtinen, Mikko Räsänen, Shadi Jansouz, Karita Peltonen, Emmi Jokinen, Jay Klievink, Keerthana Ganesh, Mari Ainola, Eva Sutinen, Mikko Rönty, Elli Narvi, Anil Thotakura, Pipsa Saharinen, Satu Mustjoki, Ilkka Ilonen, Heidi M. Haikala

**Affiliations:** ^1^ Translational Immunology Research Program (TRIMM), Research Programs Unit, Faculty of Medicine, University of Helsinki, Helsinki, Finland.; ^2^ iCAN Digital Precision Cancer Medicine Flagship, University of Helsinki, Helsinki, Finland.; ^3^ Hematology Research Unit Helsinki, Helsinki University Hospital Comprehensive Cancer Center, Helsinki, Finland.; ^4^ Department of General Thoracic and Esophageal Surgery, Heart and Lung Centre, Helsinki University Hospital and University of Helsinki, Helsinki, Finland.; ^5^ Translational Cancer Medicine Program (CAN-PRO), Research Programs Unit, Faculty of Medicine, University of Helsinki, Helsinki, Finland.; ^6^ Wihuri Research Institute, Biomedicum Helsinki, Haartmaninkatu 8, Helsinki, Finland.; ^7^ Individualized Drug Therapy Research Program, Faculty of Medicine, University of Helsinki, and Department of Pulmonary Medicine, Heart and Lung Centre, Helsinki University Hospital, Helsinki, Finland.; ^8^ Department of Pathology, University of Helsinki and Helsinki University Hospital, Helsinki, Finland.; ^9^ Immuno-Oncology, Oncology Research, Orion Corporation, Turku, Finland.

## Abstract

Despite immunotherapy’s promise in cancer treatment, patient responses vary substantially because of the individual nature of the immune system and the lack of reliable biomarkers. To address this issue, we developed a precision ex vivo platform that integrates patient-specific tumor and immune cells to study the mechanisms of antitumor immune response, predict immunotherapy outcomes, and identify effective treatments. This platform revealed unique single-cell immune response mechanisms and sensitivities to standard-of-care immunotherapies. Furthermore, we were able to identify a synergistic combination of anti–programmed cell death protein 1 (anti–PD-1) together with a Casitas B lineage lymphoma-b inhibitor that overcame anti–PD-1 resistance in selected patient samples. Activation of the interferon-γ–stimulated cytokines predicted combination efficacy, while immunosuppressive cytokines were associated with poor response. Our findings underscore the platform’s potential in tailoring immunotherapies and advancing drug development, offering avenues for personalized cancer treatment.

## Teaser

A patient-derived ex vivo platform enables the detection of unique immunotherapy responses in solid tumors.

## INTRODUCTION

Immunotherapies, especially immune checkpoint inhibitors (ICIs), have notably improved the management of various cancers by offering durable clinical benefits. ICIs targeting programmed cell death protein 1 (PD-1), programmed death-ligand 1 (PD-L1), or cytotoxic T lymphocyte–associated antigen 4 (CTLA-4) have demonstrated remarkable treatment efficacy in cancers like melanoma and non–small cell lung cancer (NSCLC) (*1*). Despite their initial success, variable efficacy and the need for more reliable biomarkers present a substantial challenge in predicting patient treatment responses to ICIs, hindering the success of immuno-oncology (IO) treatments. Despite the use of tumor PD-L1 expression as a guide for anti–PD-(L)1 therapy in NSCLC and other cancers, its dynamic nature and poor predictive capabilities have made its use as a biomarker controversial (*2*).

In addition, other immune checkpoints such as lymphocyte activation gene 3 (LAG-3), T cell immunoglobulin and mucin-domain containing 3 (TIM-3), as well as immunosuppressive cytokines, contribute to antitumor immune evasion and therapy resistance (*3*, *4*). Combining different ICIs beyond anti–PD-(L)1 and anti–CTLA-4 has emerged as a potential strategy to overcome resistance to the current IO treatments (*5*, *6*). Although several clinical studies are investigating IO combination strategies, the pace of development lags behind the emergence of new immunotherapeutic agents. Consequently, there is an urgent need for sophisticated preclinical platforms that could accurately replicate the complexity of human antitumor immunity and the tumor immune microenvironment (TIME).

The existing preclinical models for assessing IO efficacy suffer from substantial limitations. Patient-derived tumor organoids typically lack immune contexture, while humanized mice are limited by issues of reproducibility and cost. Recently, organ-on-chip (OOC) systems using tissue fragments have been used to evaluate ICI responses (*7*). Still, their applications are restricted by the small size of tumor resections and the absence of systemic immune events. Other OOC-based approaches involving inactivated or nonspecifically activated peripheral blood mononuclear cells (PBMCs), or commercially available nonmatching immune cell lines, also fail to incorporate patient-specific antitumor immunity (*8*–*12*). Therefore, there is an urgent need for a precision platform capable of faithfully mimicking personalized responses to IO drugs. Here, we describe a patient-derived ex vivo platform that leverages patient-matched tumor organoids and tumor-stimulated autologous immune cells to evaluate mechanisms of antitumor immune response and resistance mechanisms. We used single-cell methods to study the individual mechanisms of antitumor immune recognition and activation, as well as to gain better understanding of acute immune and IO drug mechanisms. OOC technology was further used to recapitulate the immune cell migration toward the tumor site and to measure immune cell–mediated tumor killing. By using this platform, we aim to accelerate the development of effective IO treatments while guiding personalized treatment selection for patients with cancer in the future.

## RESULTS

### Assessment of tumor-specific immune responses using tumor-derived organoids and circulating immune cells

To understand individual antitumor immune responses, we first generated a living biobank comprising three-dimensional (3D) organoids from lung tumor and benign tissue, achieving a successful culture rate of 83% (49 of 59) of the samples, with 15 selected for further analysis. Patient tumor characteristics, including organoid mutation profiling and diagnostic PD-L1 expression from the tumor samples, can be found in table S1.

**Fig. 1.**
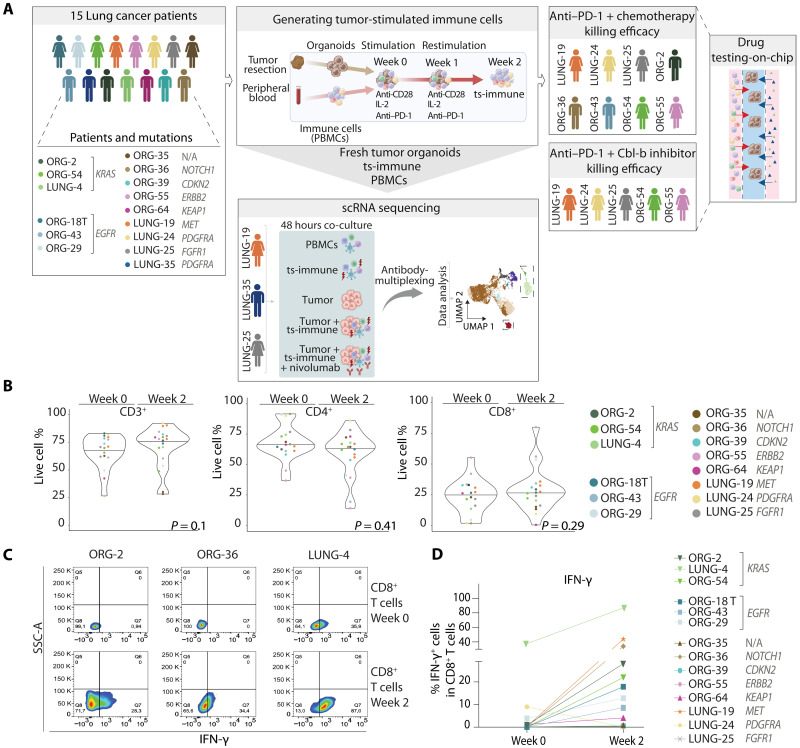
Generation of tumor-stimulated immune cells via coculture of tumor organoids and autologous PBMCs. (**A**) Experimental workflow: The study cohort included 15 patients with lung cancer carrying various mutations. Lung cancer tumor resections were used to form 3D tumor organoids. Matching peripheral blood samples were collected to isolate PBMCs, which were then cocultured with autologous tumor organoids for 2 weeks to generate ts-immune cells. These ts-immune cells were subsequently used for scRNA-seq and to evaluate immune and drug killing efficacy on a chip. The illustration indicates the source of the patient material used in the different experiments, with patients color-coded and clinically relevant mutations highlighted. (**B**) Effect of coculture on T cell subpopulations. Immune profiling by flow cytometry is presented as a violin plot, comparing the bulk percentage of viable CD3^+^ cells and subpopulations of CD4^+^ and CD8^+^ T cells before (week 0) and after (week 2) stimulation with patient-matched tumor organoids. In the violin plot, two dots of the same color represent biological replicates, while different colors indicate individual patients. The mean is depicted as a black line. The figure also includes the main clinically relevant mutations. An unpaired Student’s *t* test was used to compare the proportions of live cells at 0 and 2 weeks, with *P* values displayed in the figure. (**C**) Activation of circulating T cells by stimulation with matching tumor organoids. Representative flow cytometry plots show CD8^+^/IFN-γ^+^ lymphocytes from three patients. (**D**) Percentage of CD8^+^/IFN-γ + T cells before and after stimulation. Symbols in the graph represent the percentage of anti–IFN-γ in CD8^+^ T cells from one biological replicate (for ORG-35, ORG-54, and LUNG-19, an average of two biological replicates is depicted by each symbol). Different colored symbols indicate individual patients.

Using patient-matched PBMCs allowed us to investigate the acute and personalized interactions between non-primed immune cells and tumor-derived organoids, overcoming the limitations associated with the direct use of tumor-infiltrating lymphocytes (TILs), which may be limited in numbers and have diminished functionality. This approach also enabled systemic examination of diverse immune cell types beyond just lymphocytes. First, we cocultured the PBMCs with autologous tumor organoids, aiming to mimic systemic antitumor immune activation in the body [Fig. 1A; the protocol for generating tumor-stimulated immune cells was adapted from (*13*)]. Using flow cytometry–based immunoprofiling, we examined changes in viable T lymphocytes (CD3^+^), helper T cells (CD3^+^/CD4^+^), and cytotoxic T cells (CD3^+^/CD8^+^) in the baseline PBMCs versus in the immune cells isolated from the cocultures (tumor-stimulated immune cells, “ts-immune”). Our findings reveal that, while there was no significant overall shift in T cell proportions, individual variability was evident (Fig. 1, B and C, and fig. S1A). Notably, interferon-γ (IFN-γ) expression was induced by the tumor stimulation in 50% (7 of 14) of the samples, whereas 29% (4 of 14) were not reactive. Twenty-one % (3 of 14) of the patients had preexisting production of IFN-γ, which was further increased by tumor stimulation in two samples (Fig. 1D). IFN-γ induction in the CD8^+^ T cells was time dependent, and the dynamics varied between patients (fig. S1B), describing the individual nature of activation.

Although our study was limited in size, we observed a trend where tumor organoids with *KRAS* mutations provoked a stronger immune response than those with epidermal growth factor receptor (*EGFR*) mutations. This observation aligns with the clinically established understanding that *KRAS-* and *EGFR-*mutant tumors differ in their immunogenic potential, with *KRAS* mutations often triggering more robust immune reaction.

### Exploring individual immune responses to tumor stimulation via single-cell transcriptomics

**Fig. 2.**
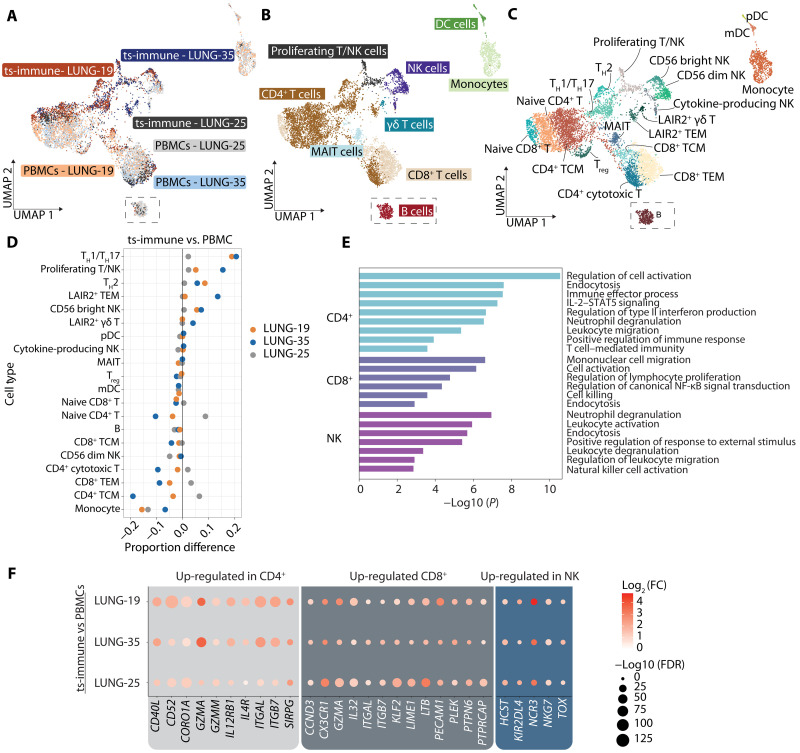
Heterogeneity of immune cell subtypes revealed by scRNA-seq following stimulation with tumor organoids. (**A**) Uniform Manifold Approximation and Projection (UMAP) visualization of sample conditions. The plot displays the clustering of PBMCs versus ts-immune cells from three patients with lung cancer, showcasing the cellular heterogeneity in response to tumor organoid stimulation. Total *n* of immune cells = 7666 (LUNG-19: *n* = 3006, LUNG-25: *n* = 2999, LUNG-35: *n* = 1661). Data integration was performed before generating the UMAP visualization. (**B**) Immune cell annotation in UMAP plot. The UMAP provides a high-level annotation of various immune cell subtypes identified in the samples. Note that B cells, originally located distantly on the plot, have been repositioned closer for clearer visualization and comparison. (**C**) Low-level immune cell annotation in UMAP plot. Twenty different immune cell clusters were identified; T_H_2, T_H_1, T_H_17, TEM, central memory T cell (TCM), T regulatory cell (T_reg_), plasmacytoid dendritic cell (pDC), and myeloid dendritic cell (mDC). (**D**) Proportional analysis of immune cell subtypes. The chart illustrates the relative proportion difference of ts-immune and PBMC in the immune cell subtypes identified in (C), a positive value indicating higher cell type proportion in ts-immune. (**E**) Pathway enrichment analysis. Bar plots display selected enrichment pathways in CD4^+^, CD8^+^, and NK cells. These pathways are based on integrated genes from ts-immune cells of the three patients following stimulation with tumor organoids. Pathway analysis was conducted using Metascape, incorporating data from KEGG, GO biological processes, Reactome gene sets, and the Hallmark gene sets. (**F**) Gene Expression Profiling. Dot plots illustrate the average expression of selected up-regulated effector genes in CD4^+^T, CD8^+^ T, and NK cells of ts-immune, in comparison to baseline PBMCs.

To better understand the underlying mechanisms of antitumor immunity and individual patient responses to tumor stimuli, we used single-cell RNA sequencing (scRNA-seq) complemented by antibody-based cell hashing to analyze circulating immune cells before and after ex vivo tumor stimulation in three patients with lung adenocarcinoma with distinct genetic mutations (Fig. 2A and table S1). The immune cells were again stimulated with the matching organoids for a period of 2 weeks, after which all cells were collected for single-cell analysis. In general, the analysis revealed a diverse array of immune cell subtypes and states, undergoing significant shifts upon tumor challenge (Fig. 2, B to D, and markers in table S2).

Specific T and natural killer (NK) cell subsets were enriched in response to the tumor stimuli, whereas naive T cells, monocytes, B cells, and dendritic cells demonstrated a relative decrease. Among the patients, most T/NK cell proliferation (indicative of tumor recognition) was observed in patient LUNG-35, followed by LUNG-19. In the same samples, pronounced T helper cell (T_H_1/T_H_17, and T_H_2) expansion together with the loss of naïve CD4^+^ population suggested increased helper T cell differentiation. LUNG-35 stood out with an increased presence of LAIR2^+^ gamma delta T cells [γδ T, CD3^+^, and T cell receptor (TCR) α/β^−^] and LAIR2^+^ T effector memory (TEM) cells following stimulation. γδ T cells are in general recognized for their antitumor capabilities, notably their ability to identify stress-induced ligands on tumor cells, bypassing the need for conventional antigen presentation (*14*). In addition, LAIR2 acts as an antagonistic inhibitor to the immunosuppressive collagen receptor LAIR1, which is known to dampen lymphocytic activity through SHP-1 signaling (*15*). This suggested an increased antitumor activation especially in patient LUNG-35.

Next, we looked at the immune cell transcriptional landscape after tumor stimulation. There were genes that showed a significant increase in expression due to stimulation in all three patients (CD4^+^ T cells: *n* = 227 genes, CD8^+^ T cells: *n* = 74 genes, NK cells: *n* = 94 genes) but also large number of individual-level changes in both up- and down-regulated genes (fig. S2, A to F). Notably, LUNG-35 and LUNG-19 shared a significant number of differentially expressed genes (DEGs) after tumor challenge, whereas LUNG-25 had more unique DEGs, emphasizing the diversity of immune responses elicited by individual tumors. By using the DEGs, we examined alterations in signaling pathways following tumor stimulation (Fig. 2E). In the CD4^+^ T cells, we found significant up-regulation in pathways related to proliferation and activation, immune response regulation, and leukocyte migration. This was accompanied by the amplification of the interleukin-2 (IL-2)/signal transducers and activators of transcription 5 (STAT5) signaling pathway, known for its role in the transcriptional response of CD4^+^ cells to TCR stimulation (*16*). Similarly, CD8^+^ T cells exhibited up-regulated pathways related to activation, proliferation, and both endocytic and cell-killing activities, suggesting a cytotoxic response. In addition, NK cells exhibited pathways related to increased activation and migration, indicating that both innate and adaptive antitumor immune responses were present.

In a further analysis of the DEGs, we identified several genes that were significantly up-regulated across immune cell subtypes. In CD4^+^ T cells, we noted an increase in the expression of *CD52*, a co-stimulatory molecule having a role in both T cell activation and suppression [Fig. 2F (*17*)]. The increased expression of *CD40LG* pointed to more robust helper T cell activation and increased T-B cell interactions, boosting antibody-mediated responses. Enhanced cell-cell communication was further suggested by the up-regulation of integrin gene *ITGAL*. In CD8^+^ T cells, the elevated levels of granzyme A (*GZMA*) and *IL32* were indicative of increased cytotoxicity and proinflammatory modulation of the immune microenvironment (Fig. 2F). Notably, the up-regulation of *PECAM1* and genes like *KLF2* and *CX3CR1* hinted at changes regarding T cell migration and interactions with endothelial cells. *CX3CR1* expression in CD8^+^ T cells has been previously suggested as a blood-based biomarker correlating with response to anti–PD-1 (*18*). Among NK cells, significant up-regulation was observed in genes including *NKG7*, *KIR2DL4*, and *TOX*, marking enhanced innate immune activation (Fig. 2F).

LUNG-25 exhibited only subtle responses within the three patients, whereas LUNG-35 displayed the most significant immune activation, followed by LUNG-19. The magnitude of the ex vivo immune response was correlating with the tumor mutational burden (TMB) observed in the organoids, with LUNG-35 having the highest TMB and LUNG-25 the lowest (fig. S2G).

### Mechanisms of lung cancer immunotherapy in tumor-immune cocultures

**Fig. 3.**
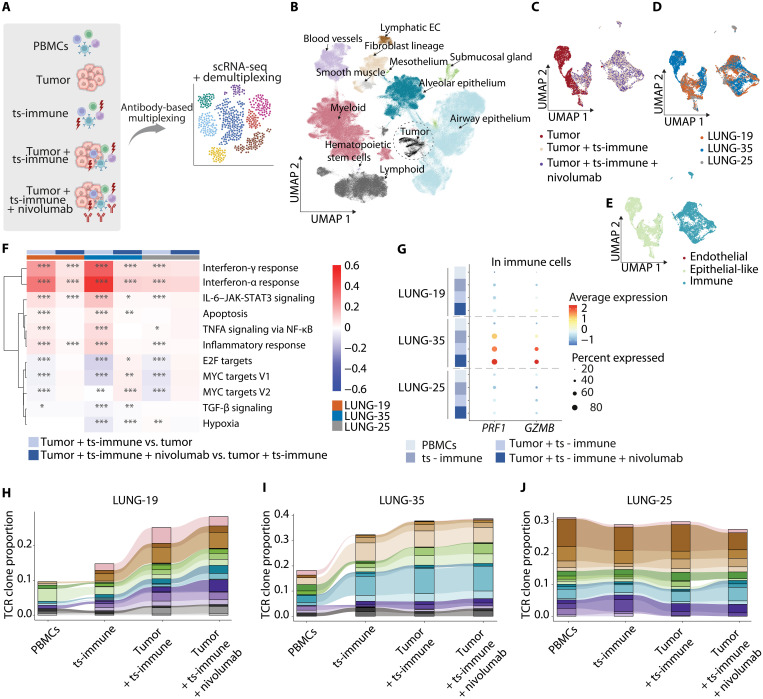
Single-cell level responses to ex vivo immunotherapy. (**A**) scRNA-seq overview. The schematic depicts the scRNA-seq process of various conditions from three patients with lung cancer. Five conditions were sequenced. PBMCs, ts-immune following 2 weeks of stimulation with autologous organoids, baseline organoids or organoids cocultured with ts-immune with or without nivolumab (100 μg/ml) for 48 hours. (**B**) Tumor cell clustering in UMAP. The UMAP plot highlights the distinct clustering of our tumor organoid cells (encircled in black with dotted lines) against healthy lung cells from the HLCA. Total number of tumor cells: 6943 (LUNG-19: *n* = 2084, LUNG-25: *n* = 1245, LUNG-35: *n* = 3614). (**C** to **E**) UMAP cell clustering of tumor and immune cell clustering from tumor alone, tumor + ts-immune, and tumor + ts-immune + nivolumab: The plots illustrate cell clustering, color-coded by the condition (C), patient identity (D), and cell type (E). The cell types were predicted using Azimuth. Data integration was not performed before generating the UMAP visualization. (**F**) Pathway analysis heatmap reveals differentially enriched pathways in different conditions. Each heatmap value denotes the difference in the average module scores. A Wilcoxon rank-sum test was performed for the single-cell level module scores, and *, **, *** signify different levels of statistical significance: FDR ≤ 0.05, FDR ≤ 0.01, and FDR ≤ 0.001, respectively. The analysis uses the Hallmark gene signature database. (**G**) Cytotoxic gene expression. The plot displays the expression levels of cytotoxic genes perforin (*PRF1*) and granzyme B (*GZMB*) in immune cells. (**H** to **J**) TCR clonality analysis. The plots show the color-coded TCR clones and their proportions in T cells from the three patients across the different conditions outlined in (A).

To further investigate the interactions between the tumor and immune cells in relation to treatment effects, we next compared isolated tumor cells to those stimulated with ts-immune, with or without anti–PD-1 treatment (nivolumab, as detailed in Fig. 3A). First, we mapped the tumor cells onto the Human Lung Cell Atlas (HLCA) reference, noting that they clustered independently, although closely approximating the epithelial cell types of the lung (Fig. 3B), similar to the observations made earlier by the HLCA project (*19*). When ts-immune cells were introduced, a distinct immune compartment emerged (Fig. 3, C to E, and fig. S3A). Unexpectedly, in sample LUNG-25, even the tumor cell-only condition displayed immune cells, indicative of the organoid model retaining some TILs (fig. S3B).

Upon tumor challenge, coculture led to an up-regulation of pathways related to interferon response, inflammation, and apoptotic signaling (Fig. 3F). This was accompanied by transcriptional changes, indicated by enhanced E2F and MYC pathway, suggesting rewiring of transcriptional programs. Immunosuppressive transforming growth factor–β (TGF-β) signaling was decreased in LUNG-19 and LUNG-35. Some of the observed changes, including the interferon signaling, were more pronounced in the presence of nivolumab. Upon addition of nivolumab, LUNG-35 demonstrated the most alterations, followed by LUNG-19, while in LUNG-25, the changes were not significant.

In LUNG-35, we also detected considerable activity of perforin (*PRF1*) and granzyme B (*GZMB*) in the T cells amplified by ex vivo nivolumab, indicating enhanced immune cell–mediated cytotoxicity (Fig. 3G). LUNG-19 also showed a slight increase in *GZMB* with nivolumab treatment, while changes were not observed in LUNG-25. In the two reactive patients, TCR sequencing revealed marked clonal expansion following nivolumab treatment (Fig. 3, H to J), indicating the presence of specific T cell clones that recognized and responded to tumor-associated antigens and treatment. When further analyzing the cell populations with the specific TCR clones, we detected a unique pattern of prevalent cell type in each clone. LAIR2^+^ TEM cells were highly significant in several TCR clones in LUNG-35 and less in LUNG-19 while not being detected in LUNG-25 (fig. S3, E to G). These findings further supported our idea that LAIR2^+^ T cell subsets could play a critical role in mediating antitumor responses also in the presence of nivolumab treatment.

### Personalized responses to the combination of nivolumab and chemotherapy revealed by a TOC platform

Clinical studies in NSCLC suggest that overall survival (OS) benefits from single-agent anti–PD-(L)1 mainly in patients with ≥50% PD-L1 expression, while patients with negative/low PD-L1 expression only occasionally benefit (*20*, *21*). However, anti–PD-1 blockade combined with chemotherapy (pemetrexed/carboplatin, PC) has shown more benefits (overall response rate, ORR: 58% in anti–PD-1 + PC and 33% in PC alone) and has become a standard-of-care treatment regardless of PD-L1 status for patients with no targeted therapy options (*22*, *23*). However, identifying the patients who will truly benefit from the treatment remains a challenge, considering the varied responses and potential for increased toxicities.

**Fig. 4.**
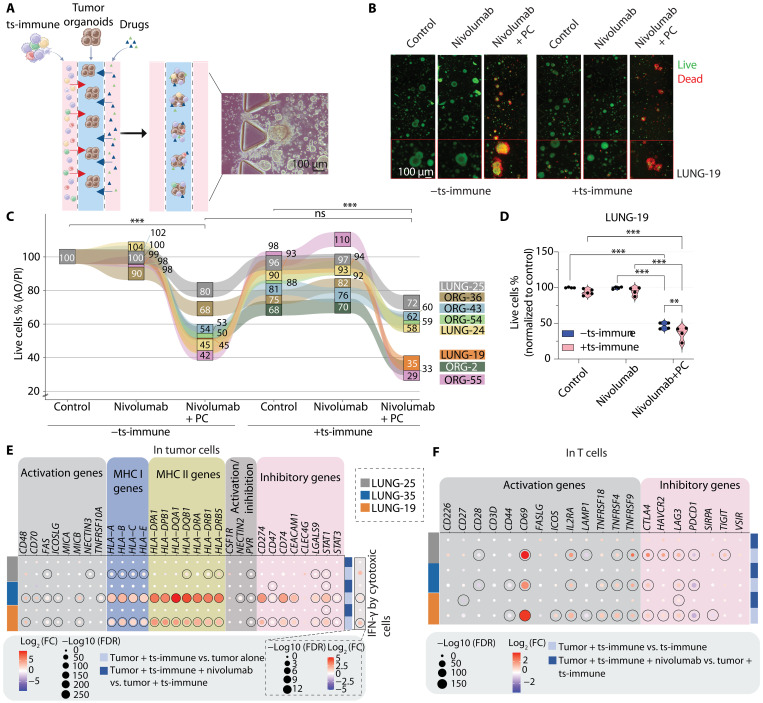
TOC models primary immunotherapy resistance and reveals differential responses to nivolumab combined with chemotherapy. (**A**) TOC design for IO testing. The chip contains a middle channel where tumor organoids are seeded in Matrigel (ECM) and separated from two side channels, where ts-immune and selected drugs were added. A bright-field image shows ts-immune infiltration to the tumor site surrounding the organoids after 24 hours of seeding. (**B**) Live (AO, green) and dead (PI, red) fluorescence images of LUNG-19 organoids with or without ts-immune and subjected to different treatments after 48 hours of coculture in the TOC. (**C**) Overview Sankey diagram showing tumor cell viability in control condition or in response to single-agent nivolumab (100 μg/ml) or its combination with 2.5 μM/375 μM PC after 48 hours of coculture in the TOC with or without ts-immune cells. Values indicated in different colored boxes or linked by differently colored lines are the means of two to five technical replicates (in LUNG-19, the mean indicates technical replicates of two biological replicates) and represent the percentages of viable tumor cells in each patient (*n* = 8). **P < 0.01; ***P < 0.001; ns, not significant, unpaired *t* test. (**D**) Live tumor cell % quantification of LUNG-19 48 hours after addition of immune cells and treatments. The median is indicated as a black line. (**E**) Dot plot showing the expression changes of activating or inhibitory checkpoints and ligand genes in tumor cells after interaction with ts-immune with or without nivolumab (100 μg/ml, 48 hours). IFN-γ expression by cytotoxic immune cells is shown. (**F**) Dot plot showing the expression of immune checkpoints and activation genes in T cells upon interaction with tumor cells with or without nivolumab (100 μg/ml, 48 hours). In (E) and (F), circled dots indicate statistical significance.

To model the individual responses to anti–PD-1 and chemotherapy more physiologically, we used a microfluidic tumor-on-a-chip (TOC) system, integrating tumor organoids, ts-immune cells, and extracellular matrix (ECM). Tumor cells were positioned into the central channel of the chip and allowed to form organoids in the ECM to mimic the tumor microenvironment (TME). The previously attained ts-immune cells were added to the proximal side channel to model immune cell infiltration into the tumor from the circulatory. Drugs were added to the distal side channel to assess the drug efficacy and distribution into the tumor (Fig. 4A). Immune cells were allowed to migrate and interact with the tumor cells for 48 hours before further analysis. Immune cell infiltration toward tumor organoids was further validated using a microwell plate, with infiltration into the organoids observed 5 hours after tumor-immune coculture (fig. S4A).

First, we used the platform to evaluate responses to nivolumab alone or combined with chemotherapy (PC) across eight patients with NSCLC from various mutational subgroups. Notably, all patients exhibited low baseline PD-L1 expression in both clinical samples and organoids (table S1), clinically associated with poor single-agent nivolumab response. Within the TOC, introducing ts-immune cells enhanced the eradication of tumor organoids, although baseline immune cell–mediated killing was highly variable among patients (Fig. 4, B to D). The addition of nivolumab alone together with the ts-immune did not significantly enhance organoid killing, which aligns with expectations for patients exhibiting low/negative PD-L1 status. However, the introduction of nivolumab in combination with PC revealed a pronounced response in 38% (3 of 8) of the individuals (ORG-2: 20%, ORG-55: 13%, and LUNG-19: 11% increase in killing efficacy compared to PC alone; Fig. 4C). LUNG-25, which showed poor immune activation and nivolumab effects in the mechanistic studies, demonstrated a lack of treatment response. In contrast, LUNG-19, characterized by high-TMB and more pronounced immune activation, was responsive. This suggests that the initial capacity for antitumor immune recognition and activation may serve as a predictive factor for the effectiveness of combining nivolumab with chemotherapy. Our findings support the clinical understanding that certain patients, including those with low PD-L1 expression, may derive substantial benefits from combining PD-1 blockade with chemotherapy. In addition, ex vivo testing could prove beneficial in identifying these individuals.

### Immune checkpoint alterations after tumor stimulation and nivolumab treatment

While we observed primary resistance to single-agent nivolumab in the PD-L1–negative/low patient samples, in the light of recent studies on IO combinations, these patients might respond more favorably to IO combination therapies involving other immune checkpoints. Consequently, we investigated the effects of immune stimulation and nivolumab treatment on checkpoints and ligands beyond PD-(L)1 (fig. S4B). In tumor cells, immune stimulation revealed an interplay of both immune activating and inhibitory signals (Fig. 4E), including up-regulation in immune-activating signals such as the *FAS* death receptor gene and the NK-activating ligand *MICB*. The induction of human leukocyte antigen genes suggested enhanced visibility of tumor cells to T cells. In addition, several inhibitory molecules, such as the TIGIT ligand CD155 (*PVR*), as well as TIM-3 ligands Galectin-9 (*LGALS9*) and CEACAM1, were also induced. We also noted an up-regulation of the IFN-γ–induced transcription factors *STAT1* and the immunosuppressive *STAT3*, also important for PD-L1 (*CD274*) transcription. The effects of nivolumab on the tumor cells were minor, with only *STAT1* showing significant up-regulation.

In T cells, immune stimulation by the tumor led to the up-regulation of many co-stimulatory receptors, including *ICOS*, *TNFRSF18*, *TNFRSF4*, and *TNFRSF9/4-1BB* (Fig. 4F). Nivolumab specifically increased co-stimulatory receptor *CD27* expression in LUNG-19. Following immune cell stimulation, an inhibitory phenotype was observed in LUNG-19 and LUNG-25, characterized by the up-regulation of TIM-3 (*HAVCR2*), with TIGIT being uniquely up-regulated in LUNG-25. CTLA-4 and LAG-3 were induced in all patients after tumor interaction. Notably, nivolumab treatment led to further overexpression of LAG-3 in LUNG-19, suggesting the potential for cotargeting strategies with LAG-3–targeting antibodies. CTLA-4, TIM-3, and LAG-3 were overexpressed in LUNG-25 following the exposure to nivolumab, although the up-regulation was not significant. Regarding NK cells, the impact of nivolumab on checkpoints and their ligands was more variable and less pronounced (fig. S4C). Our data suggested, that while many of the immune checkpoints were activated upon tumor stimulation, nivolumab only had mild impact on the well-known checkpoints.

### Up-regulation of Cbl-b E3 ubiquitin ligase induced by nivolumab treatment

Although nivolumab’s influence on the well-established extracellular immune checkpoints and their ligands appeared modest, our further analysis of the DEGs identified Casitas B-lineage lymphoma-b (Cbl-b), an E3 ubiquitin ligase, as a widely up-regulated gene across a range of immune cells. Recognized for its crucial role as an intracellular checkpoint regulating both adaptive and innate immune responses, the potential of Cbl-b in enhancing antitumor immunity has lately gained attention (*24*). Recent preclinical studies have highlighted the possibility that inhibiting Cbl-b could substantially enhance antitumor immune activities (*25*), prompting the initiation of early clinical trials to explore its viability as a target for cancer immunotherapy (NCT05107674, NCT05662397).

**Fig. 5.**
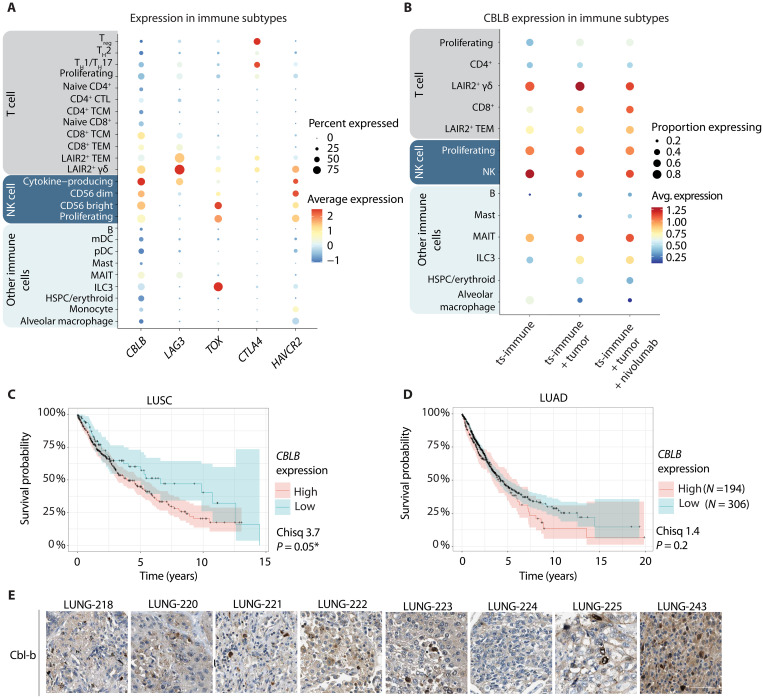
*CBLB* expression in tumor and immune cells. (**A**) Expression profile for *CBLB* across different immune cell types compared to other well-known and targetable immune checkpoints. *CBLB* was found broadly expressed in the relevant subtypes of proliferating NK cells, LAIR2^+^ cells, and effector T cells. (**B**) Expression dot plot showing *CBLB* altered by the antitumor immune stimulus and nivolumab (100 μg/ml, 48 hours). (**C**) Kaplan-Meier curve showing the correlation between low or high levels of *CBLB* expression and the survival probability of patients with lung squamous carcinoma (LUSC). The data were extracted from the Human Protein Atlas database. (**D**) Kaplan-Meier curve showing the correlation between low or high levels of *CBLB* expression and the survival probability of patients with lung adenocarcinoma (LUAD). The data were extracted from the Human Protein Atlas database. (**E**) Immunohistochemistry staining of Cbl-b expression in lung cancer tissues. Data were retrieved from the Human Protein Atlas database.

In our data, *CBLB* expression was widespread among various immune cell subsets, with a distinct and broader expression profile compared to the well-known immune checkpoints (Fig. 5A). This included high expression in the vital effector subtypes of CD8^+^ T cells and NK cells, along with the previously identified tumor-activated population of LAIR2^+^ γδ T cells. Upon nivolumab treatment, widespread up-regulation of *CBLB* was observed in CD8^+^ T cells, LAIR2^+^ γδ T cells, and LAIR2^+^ TEMs, suggesting that the most tumor-reactive immune cell subtypes were affected (Fig. 5B and fig. S5A). These findings suggest that nivolumab was inducing Cbl-b up-regulation in key antitumor immune cell subtypes, highlighting potential cotargeting strategies in the PD-L1–low/–negative patient cohort.

The clinical or cellular implications of Cbl-b expression in lung cancer remain largely unexplored. Analysis of the Cancer Genome Atlas Program expression data revealed a significant correlation between high *CBLB* expression and reduced survival in lung squamous cell carcinoma, with a similar yet nonsignificant trend observed also in lung adenocarcinoma (Fig. 5, C and D). To enhance our understanding of Cbl-b’s role in the TME, we next investigated its expression patterns to determine whether Cbl-b is predominantly localized in tumor or immune cells. Tissue sections, as well as sc-data, indicated that Cbl-b was expressed in both immune and tumor cells but with a higher magnitude in immune cells (Fig. 5E, URL: https://proteinatlas.org/ENSG00000114423-CBLB/pathology/lung+cancer, and fig. S5, A and B). As a summary, Cbl-b expression was associated with reduced survival in lung cancer, and its predominant localization in immune cells underscored its potential as a target for enhancing antitumor immunity.

### Synergistic effect of Cbl-b inhibition and nivolumab in selected patients

**Fig. 6.**
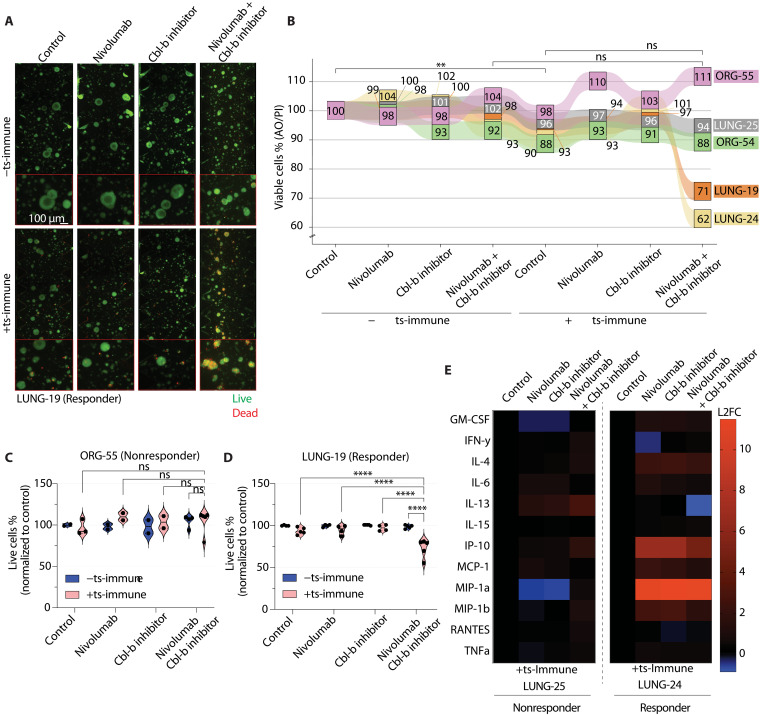
Nivolumab combined with Cbl-b inhibition improves anti–PD-1 response in a subset of patients with NSCLC. (**A**) Live (AO, green) and dead (PI, red) example fluorescence images of LUNG-19 organoids with or without ts-immune and subjected to nivolumab (100 μg/ml) or its combination with 50 nM Cbl-b inhibitor after 48 hours of coculture in the TOC. Images of control and nivolumab conditions with or without ts-immune are the same as in Fig. 4B. Scale bar, 100 μm. (**B**) Overview Sankey diagram showing tumor cell viability in control condition or in response to single-agent nivolumab (100 μg/ml) or its combination with 50 nM Cbl-b inhibitor after 48 hours of coculture in the TOC with or without ts-immune cells. Values indicated in different colored boxes or linked by differently colored lines are the means of two to five technical replicates (in LUNG-19, the mean indicates technical replicates of two biological replicates) and represent the percentages of viable tumor cells in each patient (*n* = 5). Indicated values of control and nivolumab conditions with or without ts-immune in ORG-54, ORG-55, LUNG-19, LUNG-24, and LUNG-25 are the same as in Fig. 4C. **P < 0.01; ns, not significant, unpaired *t* test. (**C** and **D**) Live tumor cell % quantification of combination treatment nonresponder (ORG-55) versus responder patient (LUNG-19). In ORG-55, scatter plots represent technical replicates, while in LUNG-19, they represent technical replicates of two biological replicates. The median is indicated as a black line. ****P < 0.0001; ns, not significant, unpaired *t* test. (**E**) Heatmap showing the secretion of 12 different cytokines in nonresponder LUNG-25 compared to the responder LUNG-24. Media were collected from the treatment conditions after 48 hours of coculture in the TOC. (*n* = 2 replicates) expressed as log_2_ fold-change (L_2_FC) relative to ts-immune control.

Given the limited response to single-agent nivolumab and the prominent up-regulation of Cbl-b upon treatment, we investigated whether Cbl-b inhibitor could enhance the response to nivolumab in the TOC model. The treatment efficacy was tested in samples of five patients with NSCLC (ORG-54, ORG-55, LUNG-19, LUNG-24, and LUNG-25). Among them, ORG-55 and LUNG-19 had previously shown an increased T cell killing response to the combination of nivolumab with chemotherapy, unlike ORG-54, LUNG-24, and LUNG-25. We found that neither single-agent nivolumab nor the Cbl-b inhibitor alone significantly increased immune cell–mediated tumor killing or caused cytotoxicity to the tumor cells in the absence of immune cells (Fig. 6, A to D). However, the combination of nivolumab and Cbl-b inhibitor demonstrated a synergistic effect, enhancing immune cell killing capability in selected patients, notably more than in conditions without immune cells. This synergy was observed with 40% (2 of 5) of the patients showing a marked benefit from the combined treatment ex vivo (LUNG-19 and LUNG-24), while 60% (3 of 5) did not respond (ORG-54, ORG-55, and LUNG-25). Notably, LUNG-19 had earlier responded positively to the combination of nivolumab with chemotherapy, suggesting broader IO benefits. Meanwhile, LUNG-25 remained unresponsive, whereas LUNG-24 showed a unique response exclusively to the combination of nivolumab and Cbl-b inhibitor.

Cytokines, as critical signaling proteins, orchestrate immune responses against tumor cells within the TME/TIME. To investigate the impact of nivolumab and Cbl-b inhibition on immune cell activity within the chip, we analyzed the secreted cytokines extracted from the TOC, comparing the cytokine profiles between a combination responder (LUNG-24) and a nonresponder (LUNG-25) model. Intriguingly, in the responder patient sample, we detected elevated levels of IFN-γ–induced cytokines, such as IP-10, MIP-1a, and MIP-1b, known to promote T cell recruitment and activation (Fig. 6E). Conversely, the nonresponder, LUNG-25, exhibited high levels of IL-13, recognized for its role in inhibiting antitumor immune response (*26*). Increases in IL-13 levels were induced by both nivolumab and Cbl-b inhibitor, but a synergistic increase was observed with their combination.

The contrast in the observed cytokine profiles may provide valuable insights into the mechanisms underlying specific antitumor responses in certain patients. In summary, our results underscore the potential of combining nivolumab with a Cbl-b inhibitor to enhance therapeutic responses in a subset of patients with NSCLC. Furthermore, this study highlights the utility of ex vivo and cocultures and OOC models in unraveling individual patient mechanisms and responses to IO strategies.

## DISCUSSION

ICIs have become the standard of care in the treatment of NSCLC. However, current ICIs benefit only a fraction of patients and lack accurate biomarkers for predicting treatment responses. This challenge is compounded by the rapid development of numerous new IO drugs and drug combinations, with an insufficient number of patients available to test their efficacy in a real-world setting. To navigate these complexities, the integration of functional precision medicine methodologies, such as personalized TOCs, emerges as a promising strategy. These approaches not only offer the potential to simultaneously evaluate multiple treatment options but also promise to expedite the preclinical phases of IO and biomarker development. While ex vivo models with immune cells have previously been established, they often lack personalization or throughput, limiting their utility for IO drug testing. Previous approaches have heavily relied on unmatched immune cells or TILs as sources of immune cells, undermining the systemic effects taking place in the cancer immunity cycle. Furthermore, recent study on lung and colorectal tumors showed that tumors can carry substantial amounts of CD8^+^ bystander TILs, whose presence might not be directly relevant to the antitumor immune response (*27*). Small clinical samples can also result in a limited yield of tumor cells and TILs for functional testing.

Here, we developed a functional ex vivo IO testing platform, which incorporates patient-matched tumor and immune cells, and allows for high-throughput testing and future inclusion of personalized TME components such as fibroblasts and endothelial cells. One of the unique features of our platform is the systemic utilization of immune cells stimulated with autologous tumor organoids rather than through nonspecific activation using antibodies. Our findings align with those of Dijkstra *et al.* (*28*), demonstrating the induction of IFN-γ production by CD8^+^ T cells in around 50% of patients following the coculturing of circulating immune cells with matching organoids. By combining stimulation assays with single-cell methods, we were able to study and identify individual antitumor immune response and resistance mechanisms at the cellular level. This approach could be especially useful for identifying previously unknown IO targets and biomarkers in the future. Specifically, we found that the activation and clonal expansion of LAIR2^+^ cells appeared to correlate with effective antitumor activity, whereas intracellular immune checkpoint Cbl-b was identified as an interesting therapeutic target.

Previous studies have shown that genetically engineered mice with *CBLB*^−/−^ T cells exhibit hyperactivated T cell and autoimmune responses (*29*, *30*). In the context of antitumor immunity, the same knockout was shown to downmodulate immune suppression and induce IFN-γ–dependent antitumor immunity (*31*). In addition, genetic inhibition of Cbl-b has been proven to prevent chimeric antigen receptor (CAR) T cell exhaustion. In our study, we observed that *CBLB* expression was widespread in multiple effector immune cell subtypes, with a notable increase in the most tumor-reactive immune cell types following nivolumab treatment. Although Cbl-b has been loosely linked to the regulatory pathways of PD-L1 (*32*), and the first-in-human studies with Cbl-b inhibitors have recently commenced (NCT05107674 and NCT05662397, the latter in combination with anti–PD-1 cemiplimab), further research is necessary to clarify Cbl-b’s precise role within the PD-1/PD-L1 signaling pathway.

Last, by using the TOC model, we successfully mimicked primary nivolumab resistance and identified potential responders to nivolumab in combination with chemotherapy. The TOC system was also used to trial the combination IO treatment using nivolumab together with Cbl-b inhibitor, which proved effective in a subset of patients. Notably, the positive responses observed with either nivolumab and chemotherapy or with Cbl-b inhibitor occurred despite the tumors exhibiting negative/low PD-L1 expression, underscoring the limitation of PD-L1 immunohistochemistry as the only pretreatment adjunct currently in clinical use. In addition, exploring therapeutic strategies that target the observed cytokines could optimize antitumor responses from ICIs, offering new tools for personalized cancer therapy. The development of “monitoring biomarkers,” derived from cytokine profiles obtained through ex vivo IO testing platforms, holds promise for advancing personalized, more effective immunotherapy treatments.

Despite the innovative approach of integrating patient-specific tumor and immune cells to predict immunotherapy outcomes, this study acknowledges certain limitations. First, the ex vivo platform may not fully recapitulate the complexity of the TME and its influence on immune cell function. In addition, the study’s findings are derived from a limited number of patient samples, which may restrict the generalizability of the results. Further co-clinical validation in a larger cohort is necessary, both for the platform and for the identified IO drug combination, to confirm the platform’s predictive value and the identified biomarkers for immunotherapy response. As a summary, we describe a patient-derived ex vivo IO testing platform that enabled the detection of synergistic combinations of anti–PD-1 therapy with Cbl-b inhibitor or chemotherapy, effectively overcoming poor responses to single-agent anti–PD-1 treatment.

## MATERIALS AND METHODS

### Reagents and resources

All used reagents and resources (including cell culture reagents, antibodies, and drugs) can be found from table S3: Reagents and resources.

### Patient samples

Patient primary tumor and blood samples were collected in accordance with the Declaration of Helsinki from consented patients with lung cancer with concurrent BioBank deposit. For the study, Helsinki University Hospital (HUS) IRB has been granted (HUS/8/2022) with a statement from the institutional ethical board HUS/970/2021. Peripheral blood was collected into lithium heparin vacuum tubes according to HUS Laboratory’s standard protocols in a laboratory chosen by the patient. Patients were recruited systematically from a tertiary hospital’s thoracic surgery service. All patients underwent a planned surgery for malignant lung tumor following a multidisciplinary team consensus meeting. Of 15 patients included in the study, 12 were treatment-naïve local primary lung cancers, 1 was primary lung cancer operated after neoadjuvant therapy, and 2 were lung metastasis of colorectal cancer who underwent metastasectomy. Furthermore, tumors with interesting oncogenic mutations (such as *EGFR*, *KRAS*, and others) were included.

### Peripheral blood lymphocyte isolation

PBMCs were isolated from peripheral blood by SepMate density gradient centrifugation according to the manufacturer’s instructions. PBMCs were then assessed for viability and cultured or cryopreserved.

### Cryopreservation of PBMCs

Isolated PBMCs were resuspended in freezing medium at a concentration of 5 to 10 × 10^6^ cells/ml and placed in cryo freezing container at −80°C for 24 hours to allow even cooling. The following day, the samples were moved to −150°C for long-term storage.

### Tumor organoid establishment and culture

Primary lung tumor surgical specimen samples were stored in wash medium and delivered to the laboratory (samples were given the codes ORG- and LUNG- since they were established in two different laboratories) within an hour since the tissue was collected. Upon arrival, a small piece of the tissue was cut and quickly put into a freezing tube to be stored at −80°C for future sequencing if required. The rest of the tissue was minced using surgical scalpels to 1 to 2 mm^3^ fragments and further dissociated with digestion medium on a shaker at 37°C overnight. The digestion was stopped by one wash with 20% fetal bovine serum (FBS) in cold Dulbecco’s modified Eagle’s medium (DMEM) and followed by two washes with phosphate-buffered saline (PBS). To increase the chance of successfully establishing tumor organoids from most of the patients and be able to isolate tumor cells and fibroblasts, we developed a two-step establishment culture system. First, we embedded half of the cells/fragments into 3D culture using Matrigel. Thirty minutes after embedding, Matrigel solidified and special organoid medium was added. Second, the other half of cells/fragments were established as 2D cultures by resuspension with standard medium and then plated in two wells of a tissue-treated six-well plate. After 4 to 6 days and on the basis of cells status following the 2D culture initiation, every well was resuspended, and suspension was transferred to the adjacent well, while the original wells were supplemented with more media. This step was repeated after 2 days with the new well ending up with six or more wells per sample. This allows for enrichment of epithelial tumor cells or fibroblasts in different wells. The fibroblasts were then collected and expanded in standard medium. Meanwhile, confluent epithelial cells at passage zero or one were collected and embedded in Matrigel allowing organoid formation.

Organoid growth was assessed with a Nikon Eclipse TS100 microscope. On the basis of their growth, the organoids were passaged once every 1 to 2 weeks at a 1:2 split ratio. Organoids were dissociated with prewarmed TrypLE express for 5 to 10 min at 37°C. To stop the TrypLE express effect, cold 20% FBS in DMEM/F12 was added and samples were centrifuged (300*g* for 5 min at 4°C), which was followed by a wash with cold advanced DMEM/F12. At this step, the organoids were broken into single cells or aggregate of three to five cells that were resuspended with Matrigel. The Matrigel suspension was domed and solidified at 37°C for 30 min and then overlaid with special organoids medium. The presence of tumor-bearing driver mutations in the organoids was validated using whole-exome sequencing.

### Organoid-PBMC coculture

For the generation of tumor-stimulated immune cells (ts-immune), we have adapted a protocol that was established by Cattaneo *et al.* (*13*). Two days before coculture, tumor organoids (1 to 5 × 10^4^ for every 1 × 10^6^ PBMC) were isolated from Matrigel by mechanical dissociation followed by 15 min of incubation with preheated dispase (2 mg/ml in PBS) at 37°C. The dispase was deactivated by 0.5 M EDTA, washed with PBS, resuspended with complete organoid media, and plated in tissue culture–treated six-well plate. Organoids were cultured for 24 hours at 37°C. One day before coculture, the organoids were stimulated overnight with IFN-γ (200 ng/ml) to enhance their antigen presentation. Ninety-six–well U-bottom plates were coated with anti-CD28 antibody (5 μg/ml; to provide co-stimulatory signals) in PBS (50 μl per well). The plate was wrapped with parafilm and incubated for 24 hours at 4°C. Cryopreserved PBMCs were thawed in T cell thawing medium followed by centrifugation. The PBMCs were then incubated for 15 min at 37°C in 5 ml of T cell thawing medium with 1:1000 benzonase and then washed with T cell thawing medium. The cells were resuspended at 2 × 10^6^ per ml in T cell medium with IL-2 (150 U/ml) and incubated overnight in a 15-ml falcon tube at 37°C.

The next day, organoids stimulated with IFN-γ were collected and resuspended with 1 ml of TrypLE Express and combined with the remaining cells that adhered to the bottom of the six-well plate for 5 min at 37°C and then washed with PBS. Dissociated organoids were then resuspended at 5 × 10^4^ cells per ml of T cell medium. Overnight-incubated PBMCs were washed with PBS and resuspended at 1 × 10^6^ cells/ml in T cell culture medium and supplemented with IL-2 (300 U/ml) and anti–PD-1 (40 μg/ml). Equal volumes of PBMCs and dissociated organoids were mixed in a ratio of 20:1. Anti–CD-28–coated 96-well plates were washed two times with PBS. Tumor cells and PBMCs were cocultured by plating 200 μl of the dissociated organoid-PBMC suspension per well. Coculture medium was refreshed three times a week including IL-2 (300 U/ml) and anti–PD-1 (40 μg/ml). Seven days after coculture, PBMCs were collected and counted and then restimulated for another 7 days with freshly isolated organoids (which had been stimulated with IFN-γ 24 hours before coculture) by seeding 2 × 10^5^ PBMCs with 10^4^ dissociated organoids per well of anti-CD28–precoated plate. By the end of coculture, ts-immune were collected and cryopreserved or used for downstream analysis and tumor killing assay. Ts-immune cells were cryopreserved at 5 × 10^6^ cell/ml in cold 10% dimethyl sulfoxide in human male serum and then placed in freezing container at −80°C and transferred to −150°C the next day.

### Evaluation of tumor reactivity using IFN-γ

For the test condition, 1 × 10^5^ of ts-immune were isolated from the 14 days of coculture, and for the negative control, 1 × 10^5^ of unstimulated PBMCs were used. Different conditions were pelleted at 330*g* for 5 min at 4°C and washed twice with fluorescence-activated cell sorting (FACS) buffer. After the washes, cell-surface staining was performed by adding 1:20 anti–CD-4–fluorescein isothiocyanate, 1:20 anti–CD-3–PerCP–Cy5.5, 1:200 anti–CD-8–V450 antibodies, and 1:1000 near-infrared viability dye in FACS buffer for 30 min. The cells were washed with FACS buffer twice, fixed and permeabilized for 20 min on ice, washed twice with 1× Perm/Wash buffer, and intracellularly stained with 1:40 anti–IFN-γ–APC in Perm/Wash buffer for 30 min at 37°C. The cells were then twice washed with Perm/Wash buffer, resuspended in FACS buffer, and analyzed by BD FACS VERSE.

### Drug testing using TOC

The AIM idenTx 3 microfluidic device was used. Tumor organoids were mechanically isolated from Matrigel and disassociated to single cells or aggregates of two to five cells by 10 to 15 min of incubation at 37°C with TrypLE. Disassociated tumor cells were resuspended at 2 × 10^3^ cells/μl with 90% cold Matrigel, and 10 μl of tumor cell Matrigel suspension was loaded to the middle channels of the AIM device. Matrigel was allowed to solidify for 25 min at 37°C followed by addition of 100 μl of special medium to the right and left top-bottom media channels. Tumor cells in Matrigel within the microfluidic chip were then allowed to form organoids for 48 hours at 37°C. To set up the Solid-IO cocultures and screen for immunotherapy responses, special medium was aspirated from the media inlets at day 2, and 100 μl of T cell medium with or without 8 × 10^4^ ts-immune cells were seeded to the inlet of the right-top channel and another 100 μl of T cell medium to the left-bottom channel of the device. This was followed by the addition of 100 μl of special medium with or without selected drugs to the inlets of the right top and bottom media channels. When all components were loaded to the microfluidic chip, the device was placed for 48 hours in a 37°C incubator on a rocker with a speed of 1 rpm and tilting angle of 14° to create a flow. The used drugs and their concentrations are nivolumab (100 μg/ml), Cbl-b inhibitor (50 nM/ml), and chemotherapy as combination of carboplatin (375 μM/ml) + pemetrexed (2.5 μM/ml).

### Coculture killing assay and multiplexed scRNA-seq readout

Tumor organoids were isolated using dispase (5 U/ml) for 10 to 15 min followed by EDTA deactivation and washes. The organoids were then resuspended with 2 ml of special medium per well and plated in a six-well plate and then incubated at 37°C and 5% CO_2_. After 48 hours, the organoids were collected, and an aliquot of the suspension was isolated and then treated with TryplE to dissociate the organoids into single cells to determine tumor single-cell count within the original organoid suspension. Tumor organoids were plated at 50 000 cells per well in 200 μl of special medium on a 48-well plate without or with ts-immune cells in T cell medium (1:4 target to effector ratio) and without or with nivolumab (100 μg/ml). For conditions with tumor organoids only, 200 μl of T cell medium was added to reach 400-μl final volume. For conditions with ts-immune, IL-2 (10 ng/ml) was added. Cocultures were then incubated at 37°C and 5% CO_2_ for 48 hours. The coculture was performed in duplicates for each condition. Experiments were done with tumor organoids, ts-immune, and PBMCs of three patients.

After 48 hours of coculture, cells from each well were dissociated into single cells using TryplE followed by two to three washes in 10 ml of PBS. Depending on viability, the cells were resuspended with 100 μl of cold washing buffer 1 or washing buffer 2, and then 10 μl of TruStain FcX blocking reagent was added, and cells were blocked for 10 min at +4°C. A unique TotalSeq-C hashing antibody was added to each sample (2 μl/2 μg per sample), and cells were incubated for 30 min at +4°C. The cells were then washed three to five times with 3.5 ml of washing buffer 1 or washing buffer 2, and then the samples were combined in cold PBS + 0.04% bovine serum albumin and proceeded to scRNA-seq.

Single-cell gene expression profiles were studied using 10x Genomics Chromium Single Cell 5’ Gene expression with Feature Barcoding technology platform. The Chromium Single Cell 5’RNAseq run, and library preparation were done using the Chromium Next GEM Single Cell 5’ Immune Profiling with Feature Barcoding technology version 2 chemistry. The sample libraries were sequenced on the Illumina NovaSeq 6000 system using the following read lengths: 26 base pair (bp) (read 1), 10 bp (i7 index), 10 bp (i5 index), and 90 bp (read 2).

## Supplementary Materials

### The PDF file includes:

Supplementary Methods Figs. S1 to S5 Tables S1 and S3 Legend for table S2 References
