# Supplementary material for: Ex vivo modeling of precision immuno-oncology responses in lung cancer
Source: Sci Adv. 2024 Oct 30;10(44):eadq6830. doi: 10.1126/sciadv.adq6830 (PMC11524168; doi:10.1126/sciadv.adq6830)
Supplement: Supplementary file 1 — Supplementary Methods Figs. S1 to S5 Tables S1 and S3 Legend for table S2 References [file sciadv.adq6830_sm.pdf]

Science Advances
AAAS

Supplementary Materials for

Ex vivo modeling of precision immuno-oncology responses in lung cancer

Bassel Alsaed *et al.*

Corresponding author: Heidi M. Haikala, heidi.haikala@helsinki.fi

Sci. Adv. **10**, eadq6830 (2024)
DOI: 10.1126/sciadv.adq6830

The PDF file includes:

Supplementary Methods
Figs. S1 to S5
Tables S1 and S3
Legend for table S2
References

Other Supplementary Material for this manuscript includes the following:

Table S2

Supplementary Methods

Cytokine profiling

Cytokine profiling was performed from the cell culture medium after 48 hours of co-culture in the platform. The profiling was performed using Bio-Plex Pro Human Immunotherapy Panel with Luminex 200 instrumentation.

Live/Dead assay

Live/Dead fluorescence staining was performed by loading 1:5 Nexcelom ViaStain™ AO/PI Staining Solution diluted in PBS. The cells were incubated with the dye for 10 minutes in the dark then washed three times with PBS. Cells were then fixed by adding 2 % PFA for 10 minutes followed by washing with PBS. Each condition was imaged with the Nikon Eclipse Ti-E microscope.

Image quantification

Images were quantified by ImageJ software. Before quantification, all data were blinded to avoid bias. The detailed developed quantification steps are as follows: **I**- open images and split color channels (Live-green and Dead-red) where each channel is treated separately, **II**- from edit choose the option conversion and select a- scale when converting, b- weighted RGB conversion, **III**- from analysis choose set measurement then select area, limit to threshold, and decimal places to 3, **IV**- convert each channel to 8-bit by selecting image, type, then 8-bit which will convert channels to gray, **V**- measuring mask was generated independently to fit the gen channel area, open the mask and place it on the area to be measured, **VI**- from image chose adjust then threshold then select “Over/Under” and set, then choose lower threshold to be 30 and upper threshold to be 255, each channel will be converted to blue and organoids/cells will remain gray, **VII**- to measure the coverage area of organoids/cell in the live and dead channel press analyze then measure each channel. Values obtained from each channel were summed up to get the total value. To get the live

and dead ratio, values from the green channel/total value x 100 = % of live organoids and values from the red channel/ total value x 100 = % of dead organoids were used.

Immune cells infiltration into organoids

Tumor cells were seeded at a density of 1.125 x 10^6 cells/mL in special medium in each well of the Sphericalplate 5D 24-well plate and incubated at 37°C for 3 days, allowing size-unified organoid formation across all micro-wells. NK cells were then labeled with a blue cell tracker dye to monitor their infiltration within tumor organoids and co-cultured with the pre-formed organoids in the Sphericalplate 5D at a 2:1 tumor-to-NK cell ratio. To assess NK cell killing, Caspase-3/7 (green fluorescence) was added, and the plate was incubated at CellCyte for live imaging over the course of 72 hours.

Single-cell data pre-processing using Cellranger

Cellranger multi v7.1.0 (*33*) was run by us to preprocess the raw sequencing files of scRNA-seq, scTCRab-seq, and cell hashing. The GRCh38 human reference genome (refdata-gex-GRCh38-2020-A, refdata-cellranger-vdj-GRCh38-alts-ensembl-7.1.0) was used for scRNA-seq and scTCRab-seq, which were downloaded from the website of 10X Genomics.

Sample demultiplexing using adapted HTODemux and outlier removal

The sample demultiplexing method HTODemux, provided by Seurat (*34*), was used to perform initial clustering with *k*-medoids. For each HTO, the cluster with the lowest expression was utilized to estimate the background expression. In this case, the two clusters with the lowest expression were employed to obtain a better estimate of the background expression, especially in cases where the first cluster has few cells. Additionally, two different parameter values were set for the positive quantile value (*pq*), one for detecting multiplets and another for detecting negatives from the

remaining cells. This allowed for better control of the number of cells considered as multiplets and negatives.

When selecting the *pq* values, the comparison of the number of predicted multiplets to the number of expected multiplets was performed to ensure reasonableness in the number of predicted multiplets. For each multiplexed dataset, the *pq* for detecting doublets was set to 0.996, and for detecting negatives, it was set to 0.8. From the cells classified as singlets, outliers were further identified using the DBSCAN R package (*35*) (v1.1-11). DBSCAN was applied separately to cells assigned to each HTO to identify the main cluster(s), first based on *t*-SNE (*36*), and then on UMAP (*37*) embeddings. Prior to performing UMAP, PCA was carried out, as recommended in the sample demultiplexing tutorial of Seurat, with the number of principal components set to the number of different HTOs. Cells outside these clusters were considered as outliers and were removed.

Removal of low-quality cells

The cells classified as singlets by our sample demultiplexing method were selected. Next, Seurat v5.0.1 was used to visualize cell-level quality metrics: the total number of UMIs, number of expressed genes, percentage of reads assigned to the mitochondrial genes (MT-%), and percentage of reads assigned to the ribosomal protein genes (RB-%). For each sample, the cells with MT-% higher than 15% were removed.

Further low-quality cell populations were identified based on Seurat's scRNA-seq data analysis workflow. Normalization was performed using the LogNormalize method, the top 2000 of the most highly variable genes were selected using the FindVariableFeatures function, PCA was performed (30 components were used), the PCA embeddings were clustered using Seurat's graph-based algorithm (*38*), a two-dimensional visualization of the data was generated using UMAP, and gene markers for the clusters were identified using the FindAllMarkers function. It was observed that the UMAP visualization included cell populations with abnormally low RB-% levels, high MT-% levels, or high expression of genes related to cell stress (*39-40*). The problematic cell populations were removed, and the workflow was iterated until problematic cell populations could no longer be identified.

ScDblFinder v1.12.0 (*41*) was used to identify heterotypic doublets by running it for each sample separately. By comparing the gene expression profiles of the cells classified as doublets and singlets by scDblFinder, it was concluded that no clear heterotypic doublets were identifiable in any of the samples. Therefore, no doublets were removed with scDblFinder.

Segregation of epithelial-like tumor cells and immune cells

The basic Seurat cell type identification workflow was run for the three conditions that contain tumor cells. The data were normalized using the LogNormalize method, the 2000 most highly variable genes were selected, PCA with 30 principal components was used to reduce the dimensionality, UMAP was employed to visualize the data, and graph-based clustering (*41*) was utilized to partition the data into clusters. Azimuth v0.4.6.9002 (*42*) with the Human Lung Cell Atlas (HCLA) (*19*) as a reference was used to perform automated cell type assignment.

In the cell type assignment performed by Azimuth for the tumor-only condition, 89% of the cells were assigned as epithelial cells. However, in the Lung-25 organoid, 540 cells from the lymphoid lineage expressed T cell markers (*CD3D*, *CD3E*) and were classified as immune cells. In the other two tumor cell conditions, with tumor and immune cells combined, it was predicted by Azimuth that 76% of the cells belonged to the lymphoid lineage and 24% were epithelial cells. Based on Seurat's clustering and Azimuth's cell type assignment, each of the three tumor-cell-containing datasets was split into two separate subsets with immune and epithelial-like cells.

A total of 21812 cells were deemed to have passed the quality control, excluding the putative TILs found from the condition with only tumor cells. Out of these 21812 cells, 14869 were identified as immune cells and 6943 were identified as tumor cells.

Automated cell type assignment for immune cells

Cell type labels for the immune cells in the four immune-cell-containing conditions were assigned in an automated manner using three tools: Azimuth v0.4.6.9002 (*42*), Celltypist v1.5.2 (*43*) , and SingleR v2.2.0 (*44*). The Human PBMC Reference (v1.0.0)

and Immune v2 datasets were used as references in the prediction models with Azimuth and Celltypist, respectively. For SingleR, the Human Primary Cell Atlas (*45*), Encode (*46*), and Blueprint datasets (*47*) were used as references.

Unsupervised cell type assignment for immune cells

The cells belonging to the PBMC and ts-immune conditions (excluding the tumor-cell-containing conditions) were selected, and unsupervised cell type assignment was performed using Seurat. The samples, each with a unique patient-condition combination, were defined as the batch variable, and the "Streamlined data integration" tutorial of Seurat v5 was followed to integrate the data. The 2000 most highly variable genes were selected, and dimensionality reduction was performed using PCA with 50 principal components. To ensure that the resulting clusters were not solely driven by T cell clonality genes, the TCR variable genes, i.e., genes whose names start with *TRAV*, *TRBV*, *TRGV*, or *TRDV*, were excluded from the highly variable genes (*48*). The scVI (*49*) and Harmony (*50*) data integration methods were tested, and the results were found to be largely similar in terms of the identifiable cell populations in the UMAP plots. Harmony was chosen as the integration method because more pronounced clusters were provided for some key T cell subtypes.

Based on our examination of the UMAP plot, the resolution of Seurat's graph-based clustering was adjusted to a high initial number (3.0) to perform overclustering. Some neighboring clusters were then combined, which were considered based on gene markers to be part of the same cell type. The clusters were annotated based on gene markers of the clusters and annotations predicted by the supervised cell type identification methods Azimuth, SingleR, and Celltypist.

The automated predictions for gamma delta (γδ) T cells were discordant. They were labeled as γδ T cells since they were CD3+ but lacked a TCR with alpha and beta chains. Furthermore, *TRDC*, *TRDV2*, *TRGV1*, *and* TRGC1 were significantly upregulated in γδ T cells, supporting their classification as γδ T cells. Several killer-cell immunoglobulin-like receptor (KIR) genes (*KIR2DL3*, *KIR3DL1*, *KIR2DL1*) were also expressed in γδ T cells, which has been shown in previous studies (*51-52*).

The same unsupervised cell type assignment was then performed for all immune cells, excluding the small number of TILs found in the tumor-only condition. In addition to the PBMC and ts-immune conditions mentioned above, this also included the immune cells isolated from the two conditions that involved both tumor and immune cells. The cell types identified in this analysis were largely similar to those found in the PBMC & ts-immune analysis. However, a few additional rare cell types were identified in the analysis: type 3 innate lymphoid cells (ILC3), alveolar macrophages, and mast cells, which were most likely tissue-resident cells from the tumor tissue (fig. S3D).

Differential expression analysis

To find differentially expressed (DE) genes between the cell types, the FindAllMarkers function of Seurat was used along with the Wilcoxon rank-sum method. To enable detection of weak signals, logfc.threshold was set to 0 and min.pct was set to 0. The p-values were corrected using the Benjamini-Hochberg procedure (*53*).

The FindConservedMarkers function of Seurat was used to find cell-type-specific DE genes between the conditions, with the grouping variable set to patient. The DE analysis was thus performed between the conditions for each patient separately.

Mapping tumor cells to Human Lung Cell Atlas

The scArches (*54*) transfer learning model of HCLA (*19*) was employed to study the epithelial-like tumor cells of the three tumor-cell-containing conditions in more depth. Architectural surgery was performed on the HCLA core model, which consisted of healthy lung cells from different public datasets, using the tumor cells from our study as query. scArches enables the annotation of query cells, but it also enables the discovery of aberrant cell states based on how the query cells cluster with the reference cells in the joint UMAP embeddings. The scArches analysis was performed by following the “Mapping data to the Human Lung Cell Atlas for joint analysis and cell type label transfer” Python notebook, which was accessed from the GitHub page of HCLA.

Cell cycle scoring

The "Cell-Cycle Scoring and Regression" vignette of Seurat was used to perform cell cycle scoring for the scRNA-seq data (*55*). The clusters with high G2/M or S scores were labeled as proliferating cells. As noted in the vignette, cells expressing neither score are likely not proliferating and are in the G1 phase.

Single-cell TCR analysis

The scRNAab-seq data were analyzed using scRepertoire v1.8.0 (*56*). The analysis was run following the "Starting work with scRepertoire" Bioconductor vignette. The top 10 most expanded clonotypes based on the amino acid sequence of the beta chain in the CDR3 region were defined, which were used to compare the expanded clonotypes across the different conditions for each patient. The alluvial visualization function included in the scRepertoire R package was used to visualize clonotype expansion.

Pathway analysis

For the ts-immune vs. PBMC comparison, pathway analysis for CD4+ T cells, CD8+ T cells, and NK cells was performed using the Metascape web tool (*57*). Genes with log2FC ≥ 1 and FDR ≤ 0.05 were included, and the remaining genes were ranked based on the average log2FC calculated across the patients. The top 200 genes were selected as input to Metascape. The hallmark gene set collection from the Molecular Signature Database (MSigDB) (*57*), KEGG (*58*), and Reactome (*59*) were used as pathway databases.

While studying gene expression changes in tumor cells between the different conditions, pathway analysis based on gene program (module) scores was performed. The module scores were generated using the AddModuleScore function of the Seurat scRNA-seq analysis toolkit. All tumor cells were pooled together, and the module scores for each of the 50 pathways in the hallmark gene set collection were calculated. The statistical significance of the enrichment score changes between the conditions was assessed using the Wilcoxon rank-sum test. Multiple comparison adjustment of the *p*-values was performed using the Benjamini-Hochberg procedure.

Variant calling from whole exome sequencing

The Mutect2 method, which is part of the GATK v 4.4.0.0 software (*60*), was used to detect somatic mutations from the tumor organoids. The Best Practices Workflow for pre-processing was followed to produce the Analysis-Ready Read files. Somatic variant calling was then performed in tumor-only mode. The gnomAD germline sequencing cohort (*61*) was used as the germline resource, and the VCF file ("af-only-gnomad.hg38.vcf.gz") containing the gnomAD allele fractions was downloaded from the public-data-broad-references Google Cloud bucket. The FASTA file ("Homo_sapiens_assembly38.fasta") of the hg38 reference genome and the Panel of Normal (PoN) resource ("1000g_pon.hg38.vcf.gz") were downloaded from the same bucket. The analysis was limited to the target intervals of the KAPA HyperExome kit, as specified in the manufacturer's BED file. ANNOVAR (*62*) was used to annotate the variants with COSMIC v98 somatic cancer mutation (*63*) and AlphaMissense databases (*64*).

Tumor mutational burden

The tumor mutational burden (TMB) was defined as the number of somatic mutations per megabase of investigated genomic sequence (*65*). More specifically, the number of somatic mutations was determined by the number of mutations in the VCF file with value PASS in the FILTER column, which were predicted by the tumor-only Mutect2 pipeline. The size of the investigated genomic sequence was calculated as the total length of the intervals in the targeted regions BED file of the KAPA HyperExome kit in megabases.

Supplementary Figures

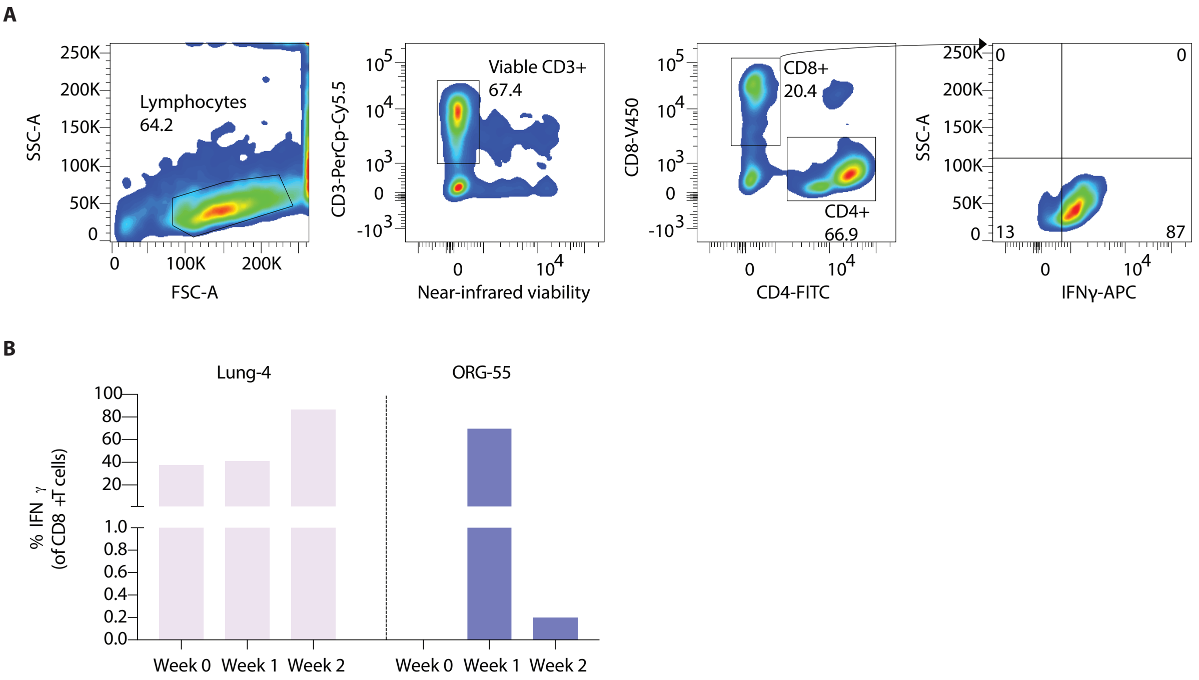

Fig. S1: Flow cytometry evaluation of CD8+ T cells stimulation (related to Fig. 1). (**A**) Following two weeks of co-culture with matching tumor organoids, T cells were evaluated for their stimulation by measuring their expression of intercellular IFN-γ. (**B**) Bar plot showing the expression of IFN-γ by CD8+ T cells in two different patients (LUNG-4 and ORG-55) at the baseline (week 0), after one and two weeks of stimulation co-culture with matching tumor organoids.

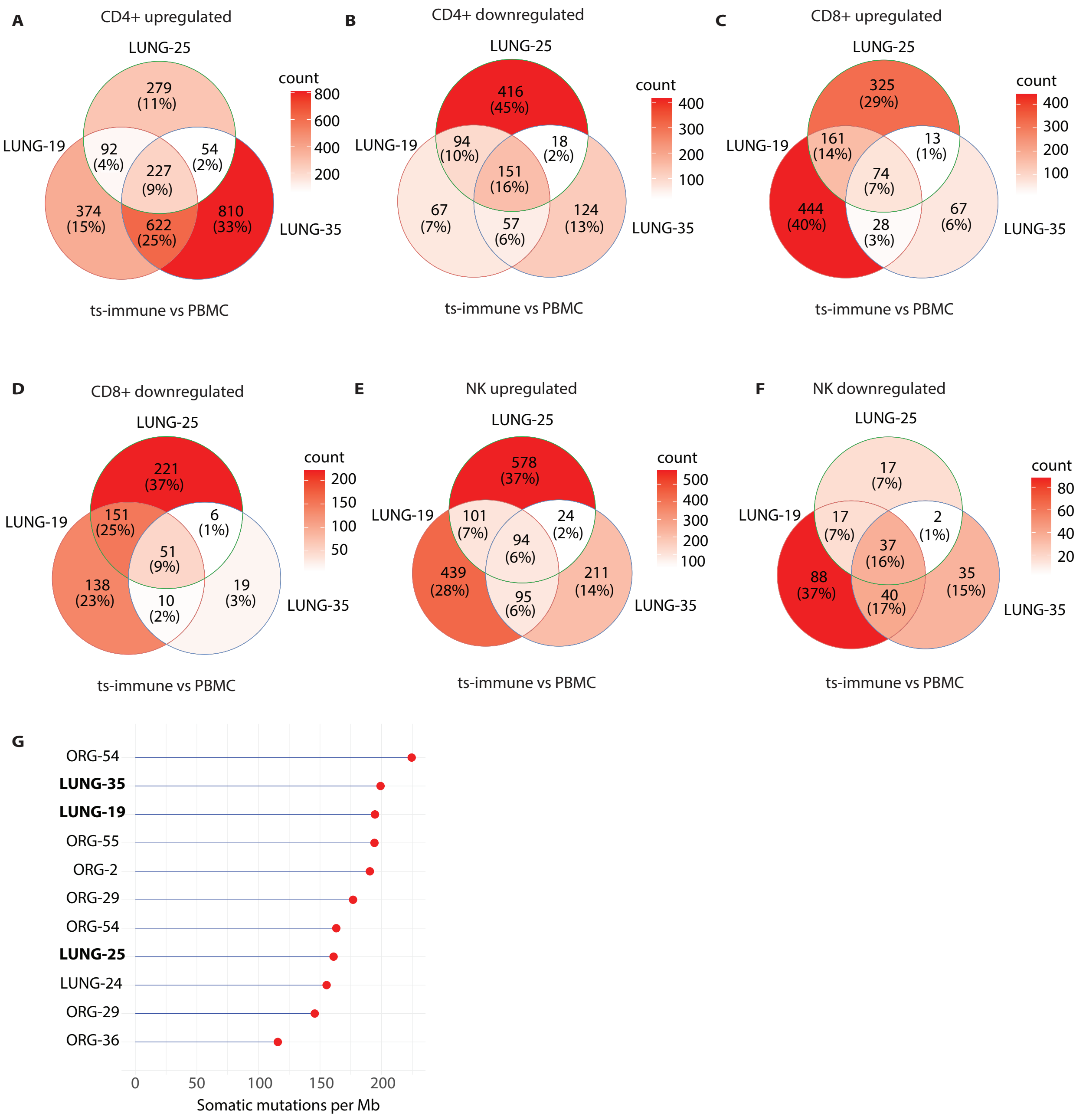

Fig. S2: Up- and down-regulated genes in the major immune cell subtypes following tumor stimulation (related to Fig. 2). (**A to F**) Venn diagram visualizing the overlap of upregulated and downregulated genes from comparisons between ts-immune and PBMC cells. The statistical analysis was performed using the Wilcoxon rank-sum test, and the p-values were corrected using the Benjamini-Hochberg procedure (FDR 0.05, log2 fold-change 0.5). (**G**) Tumor mutational burden in organoids. The values were calculated from Whole Exome Sequencing data using Mutect2 software.

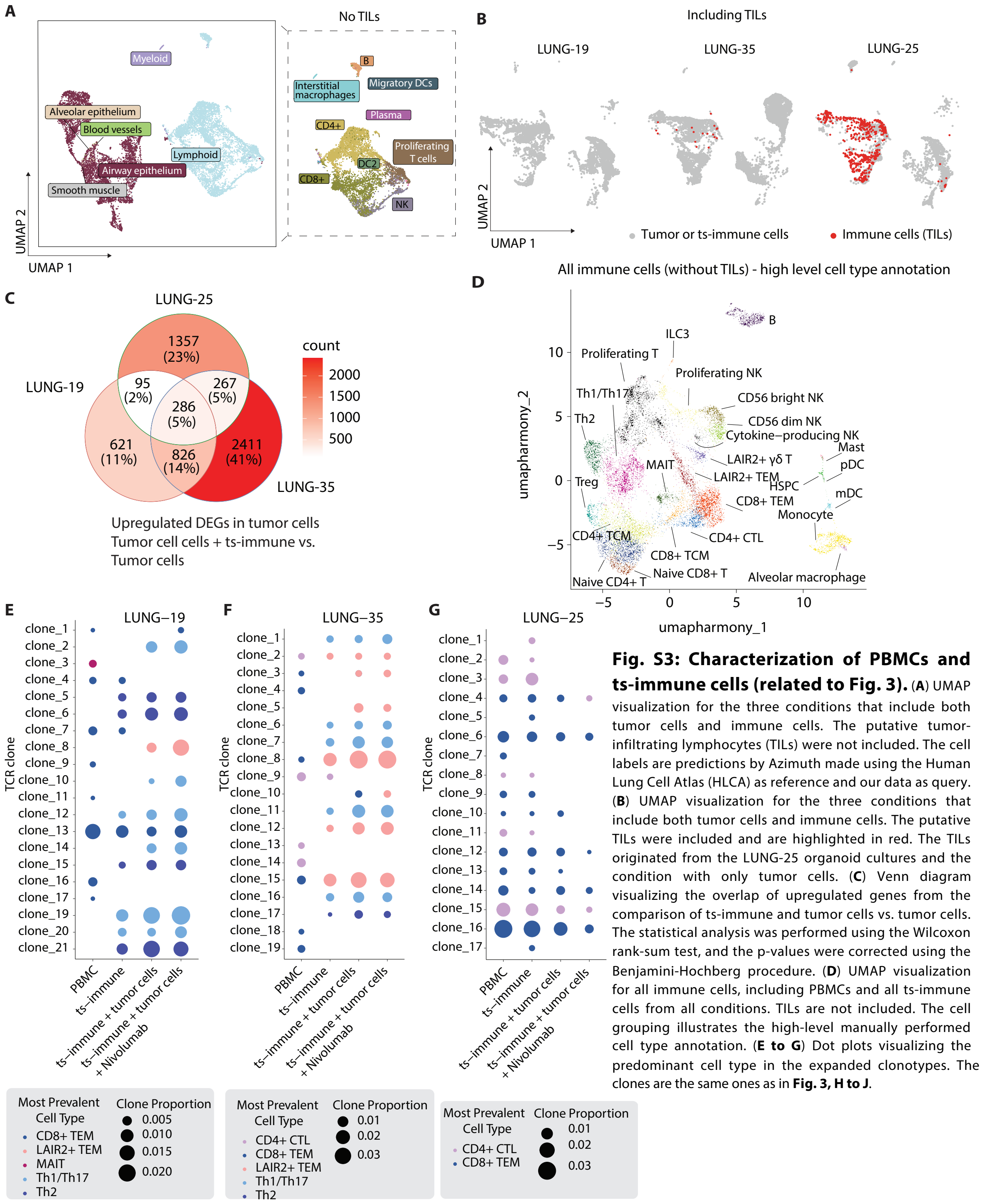

Fig. S3: Characterization of PBMCs and ts-immune cells (related to Fig. 3). (**A**) UMAP visualization for the three conditions that include both tumor cells and immune cells. The putative tumor-infiltrating lymphocytes (TILs) were not included. The cell labels are predictions by Azimuth made using the Human Lung Cell Atlas (HLCA) as reference and our data as query. (**B**) UMAP visualization for the three conditions that include both tumor cells and immune cells. The putative TILs were included and are highlighted in red. The TILs originated from the LUNG-25 organoid cultures and the condition with only tumor cells. (**C**) Venn diagram visualizing the overlap of upregulated genes from the comparison of ts-immune and tumor cells vs. tumor cells. The statistical analysis was performed using the Wilcoxon rank-sum test, and the p-values were corrected using the Benjamini-Hochberg procedure. (**D**) UMAP visualization for all immune cells, including PBMCs and all ts-immune cells from all conditions. TILs are not included. The cell grouping illustrates the high-level manually performed cell type annotation. (**E to G**) Dot plots visualizing the predominant cell type in the expanded clonotypes. The clones are the same ones as in **Fig. 3, H to J**.

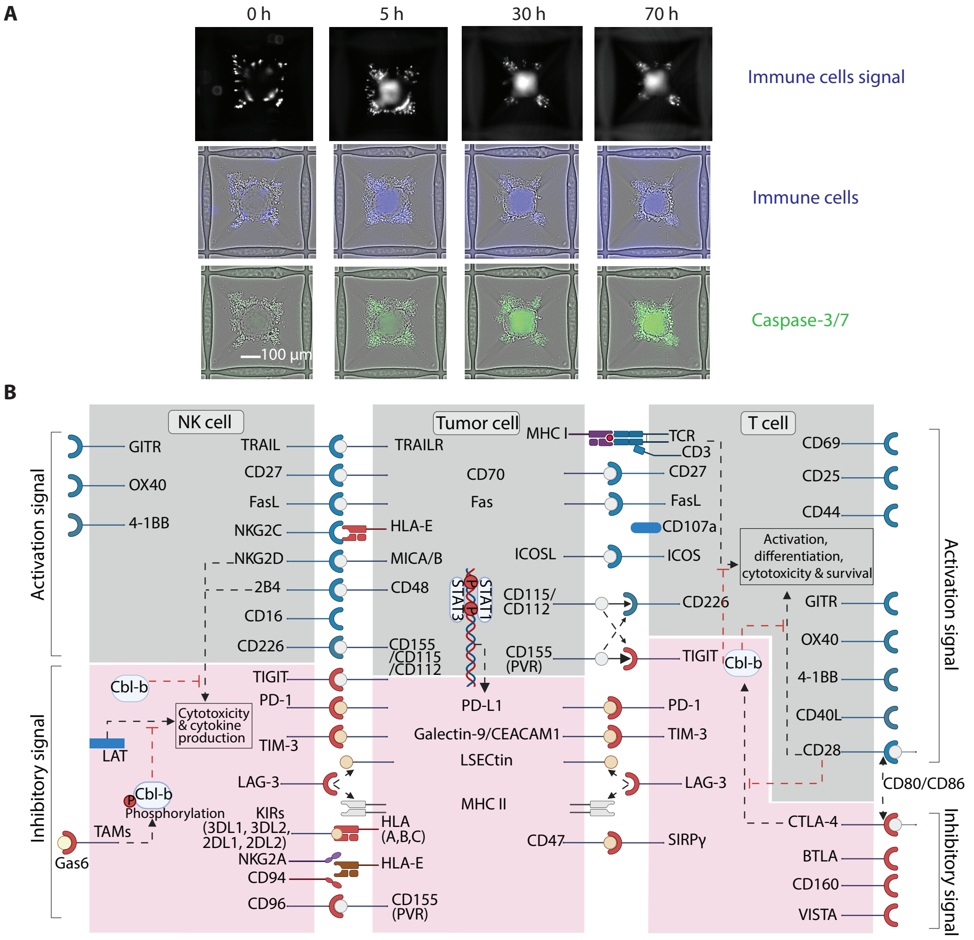

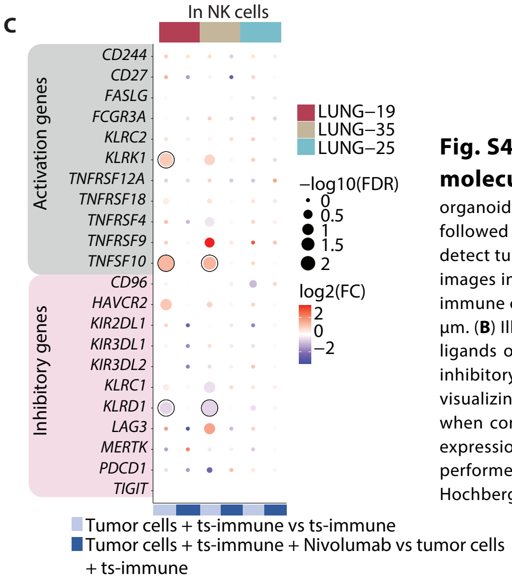

Fig. S4: Immune cell's signal and activating- inhibitory immune-related molecules (related to Fig. 4). (**A**) Image panel indicating immune cell infiltration into the organoid core. Size-unified tumor organoids were formed in micro-wells of the Sphericalplate 5D, followed by co-culture with blue-labeled immune cells and the addition of Caspase-3/7 (green) to detect tumor killing within 72 hours. Images were acquired using the live imager CellCyte. Grayscale images indicate immune cell signals, brightfield images with blue fluorescence indicate pre-labeled immune cells, and brightfield images with green fluorescence indicate tumor death. Scale bar is 100 µm. (**B**) Illustration showing intra- and extra-cellular immune check points in T- and NK cell and their ligands on tumor cells. The checkpoints are color-coded and characterized as activating (blue) or inhibitory (red) upon interaction with their ligands either from tumor or dendritic cells. (**C**) Dot plot visualizing the log2 fold-change (log2FC) and False Discovery Rate (FDR) values for different genes when comparing gene expression levels in NK cells between different conditions. The differential expression analysis was performed within each patient separately. The statistical analysis was performed using the Wilcoxon rank-sum test, and the p-values were corrected using the Benjamini-Hochberg procedure.

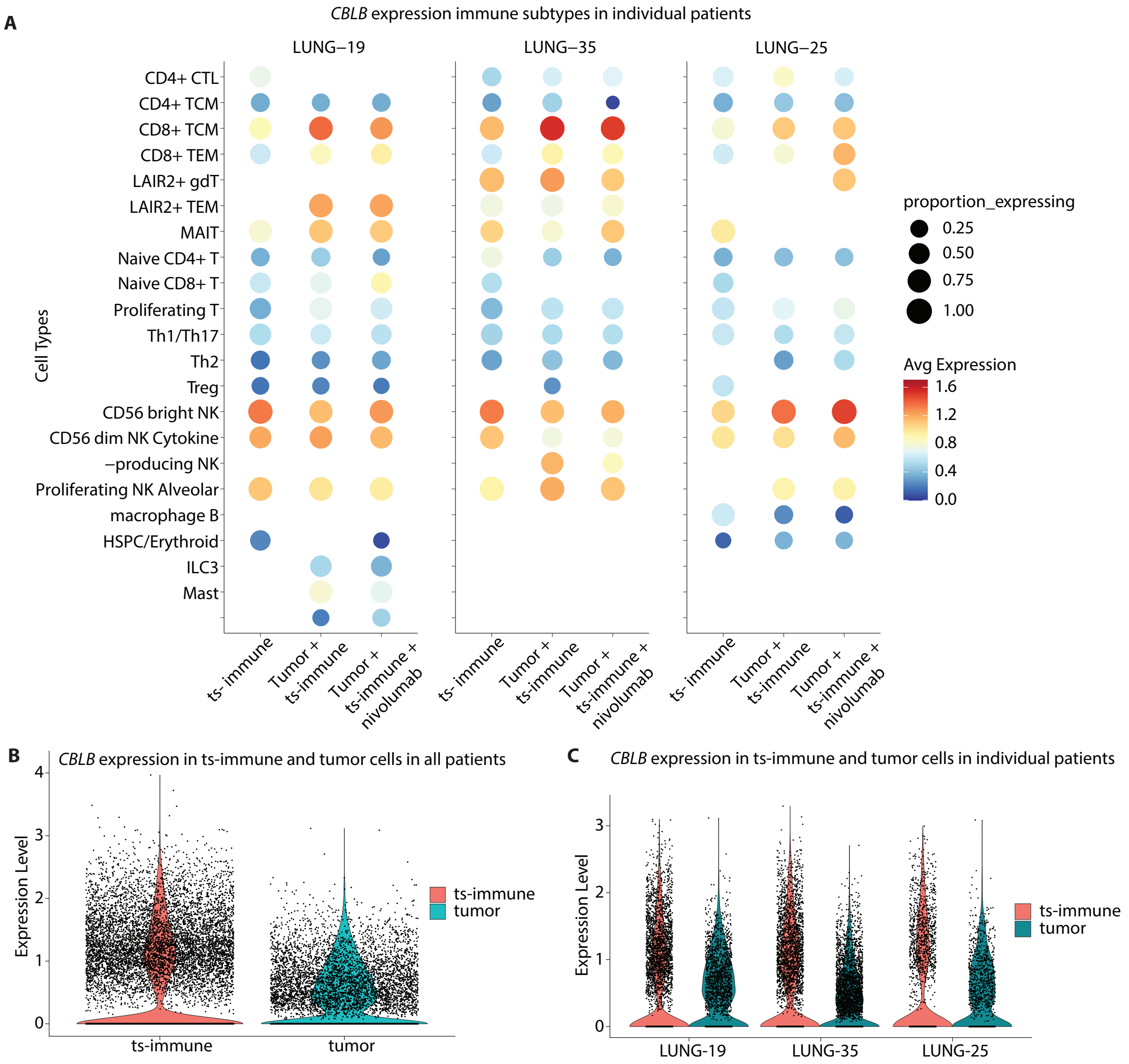

Fig. S5: *CBLB* expression in tumor and immune cells (related to Fig. 5). (**A**) Expression dot plot showing CBLB expression in the immune cell subtypes of the three patients. (**A and C**) Expression level of CBLB in scRNA-seq data from the three patients combined (**B**) or at the individual level (**C**). The violin plots show the expression level at single-cell level grouped by the classification of cells into immune and tumor cells.

Table S1. Patient characteristics for the study.

Patient	Sex	Age	Smoking	Tumor	Treatment naïve sample	Pathogenic mutations	Clinical PD-L1 expression (%)
ORG-2	M	59	C	Adenocarcinoma	Yes	*KRAS G12C; KEAP1 C288F*	15
ORG-18	F	72	N	Adenocarcinoma	Yes	*EGFR exon19del* *	0
ORG-29	F	66	E	Adenocarcinoma	Yes	*EGFR L858R*	<5
ORG-35	F	69	N	Adenocarcinoma	Yes	N/A*	N/A
ORG-36	M	81	E	Adenocarcinoma	Yes	*NOTCH1 F993C/ N994K; ROS1 A264P*	0
ORG-39	M	66	E	Adenocarcinoma	No (Cisplatin and Vinorelbine)	*CDKN2A stop-gain; TP53 A159P*	0
ORG-43	M	79	E	Adenocarcinoma	Yes	*EGFR exon19del* *	0
ORG-64	M	61	E	Epidermoid carcinoma	Yes	*KEAP1 C288F; CDKN2A; TP53 A159P*	N/A

ORG-54	F	79	E	Adenocarcinoma metastasis from colorectal cancer	No	*KRAS G12S; TP53 R248Q; KRAS 505C*	N/A
ORG-55	F	64	E	Adenocarcinoma	Yes	*ERBB2 C299S; NOTCH1 C505W; IDH1 I330F; ALK G925A*	5
LUNG-4	M	77	E	Adenocarcinoma	Yes	*KRAS (G12A)**	0
LUNG-19	F	60	C	Adenocarcinoma	Yes	*MET T922I/P664A; CDK12 R340W*	0
LUNG-24	F	59	C	Adenocarcinoma metastasized from colorectal cancer	No (Capecitabine and Radiation Therapy)	*PDGFRA D836V/F837S KIT G565V; MET P664A; RET T929I*	N/A
LUNG-25	F	74	N/A	Adenocarcinoma	Yes	*FGFR1 M217T;*	2

						MSH2 G520R/L709S	
LUNG-35	M	65	N/A	Adenocarcinoma	Yes	*PDGFRA G838S/K700N; MET G667A; PMS L718P; MET P664A; ROS1 W505R; MLH1 G198E; BRIP1 D153H*	0

F, female; M, male; C, current smoker; E, ex-smoker; N, never-smoker; and N/A, not-applicable.

**Mutation was detected in the clinical panel / organoid model was not sequenced to date.*

Table S3. Reagents and resources

REAGENT and RESOURCE	SOURCE	IDENTIFIER
**Biological Samples**		
Patient tumor lung tissue	HUS	Study HUS/8/2022
Patient blood	HUS	Study HUS/8/2022

Peptides, recombinant proteins, and chemicals		
R-Spondin 1	PeproTech	Cat#120-38
Human recombinant EGF	Invetrogen	Cat# MA5-16360
B27 supplement	Gibco	Cat#17504044
Rho kinase inhibitor Y-27632	Selleckchem	Cat# S1049
Human recombinant FGF-7	PeproTech	Cat#100-19
Human recombinant FGF-10	PeproTech	Cat#100-26
Noggin	PeproTech	Cat#120-10C
A83-01	Merck	Cat#SML0788-5MG
SB202190 (p38 inhibitor)	MedChemExpress	Cat#HY-10295
N-acetylcysteine	Merck	Cat# A9165-5G
Nicotinamide	Merck	Cat# N0636-100G
Primocin	Invivogen	Cat# ant-pm-05
N2 supplement	Gibco	Cat#17502048
Human recombinant FGF-2	R&D systems	Cat#233-FB
Ultra-glutamine I	Lonza	Cat#BE17-605E/U1

L-glutamine	Gibco	Cat#25030024
RPMI 1640	Gibco	Cat# 31870025
Human serum from human male plasma (Type AB)	Merck	Cat#H4522-100ML
Penicillin/Streptomycin	Gibco	Cat#15140122
Advanced DMEM/F-12	Gibco	Cat#12634028
Matrigel Growth factor reduced basement membrane matrix	Corning	Cat#11553620
Dispase I	Sigma Aldrich	Cat#D4818-2MG
FBS, fetal bovine serum, heat inactivated	Gibco	Cat#10500064
Trypan Blue 0.4% solution	Gibco	Cat#15250061
UltraPure™ 0.5M EDTA, pH 8.0	Invitrogen	Cat#15575-038
Human recombinant Interferon gamma	PreproTech	Cat#300-02
Interleukin-2	PreproTech	Cat#SRP3085-50UG
DMSO	Merck	Cat#D8418-250ML
Benzonase 5000 UNITS	Merck	Cat# SRP3085-50UG

PBS	R&D system	Cat#4870-500
Ficoll-Paque	Cytiva	Cat#17144003
Nexcelom ViaStain™ AO/PI Staining Solution	Fisher Scientific	Cat# CS2-0106-5ML
Human TruStain FcX™	BioLegend	Cat# 422302
Red blood cell lysis buffer (10x)	BioLegend	Cat# 420301
Trypsin-EDTA,phenol red	Gibco	Cat# 25300062

Antibodies

Mouse anti-human CD28	eBioscience	CD28.2; Cat# 14028982
Mouse anti-human CD3 (PerCP-Cy5.5-conjugated)	BD	SK7; Cat#332771
Mouse anti-human CD4 (FITC-conjugated)	Stemcell technologies	OKT4; Cat#60016FI.1
Mouse anti-human CD8 (V450-conjugated)	BD	RPA-T8; Cat#560347
Mouse anti-human IFNγ (APC-conjugated)	BD	B27; Cat#562017
Mouse anti-human CD3 (PerCP-Cy5.5 conjugated)	BD	SK7; Cat#332771

ImmunoCult Human CD3/CD28/CD2 T cell activitor	StemCell	Cat# 10970
mouse anti-human IgD (APC-conjugated)	BD	IA6-2; Cat# 561303
TotalSeq™-C0251 anti-human Hashtag 1 Antibody	Biolegend	Cat# 394661
TotalSeq™-C0252 anti-human Hashtag 2 Antibody	Biolegend	Cat# 394663
TotalSeq™-C0253 anti-human Hashtag 3 Antibody	Biolegend	Cat# 394665
TotalSeq™-C0254 anti-human Hashtag 4 Antibody	Biolegend	Cat# 394667
TotalSeq™-C0255 anti-human Hashtag 5 Antibody	Biolegend	Cat# 394669
TotalSeq™-C0256 anti-human Hashtag 6 Antibody	Biolegend	Cat# 394671
TotalSeq™-C0257 anti-human Hashtag 7 Antibody	Biolegend	Cat# 394673
TotalSeq™-C0258 anti-human Hashtag 8 Antibody	Biolegend	Cat# 394675
TotalSeq™-C0259 anti-human Hashtag 9 Antibody	Biolegend	Cat# 394677
TotalSeq™-C0260 anti-human Hashtag 10 Antibody	Biolegend	Cat# 394679
TotalSeq™-C0262 anti-human Hashtag 12 Antibody	Biolegend	Cat# 394683

Drugs

Nivolumab (anti-PD1)	Selleckchem	Cat# A2002

Cbl-b inhibitor	Synthesized by Aurigene Oncology Limited	
Carboplatin	Sigma Aldrich	Cat# C2538-100MG
Pemetrexed disodium heptahydrate	Sigma Aldrich	Cat# SML1490-10MG
**Commercial assays & reagents**		
Live/dead fixable near-IR dead cell stain kit	Invitrogen	Cat#L10119
Cell tracker (TM) blue CMAC	Fisher Scientific	Cat#C2110
CellEvent^{TM} Caspase-3/7 Green	Invitrogen	Cat#R37111
Fixation/Permeabilization solution kit	BD	Cat#554714
SepMate-50 (IVD) PBMC isolation tubes	StemCell technologies	Cat#85460
Sphericalplate 5D® in 24-well format	SP5D, Kugelmeiers Ltd, Erlenbach	
**Microfluidics chips**		
AIM idenTX 3 Chip	AIMbiotech	Cat#DAX01-PAK

Cell culture media and buffers

Medium	Components
Standard medium	Advanced DMEM/F12 supplemented 1% Ultraglutamine, 1% Penicillin/Streptomycin, 20 ng/mL FGF-basic, 50 ng/mL EGF, 5µM Y-27632.
Special organoid medium	Advanced DMEM/F12 supplemented with 500 ng/mL R-spondin, 25 ng/mL FGF-7, 100 ng/mL FGF-10, 100 ng/mL Noggin, 500 nM A83-01, 5µM Y-27632, 500 nM SB202190, 1x B27 supplement, 1.25 mM N-acetylcysteine, 5 mM Nicotinamide, 1% Ultraglutamine I, Hepes buffer 10 mM, 1% Penicillin/Streptomycin, and 50 µg/mL Primocin.
T cell-thawing medium	RPMI 1640 supplemented with 1% Ultraglutamine I, 1% Penicillin/Streptomycin, and 10% FBS
T cell-culture medium	RPMI 1640 supplemented with 1% Ultraglutamine I, 1% Penicillin/Streptomycin, and 10% human male serum.
Digestion medium	Advanced DMEM/F12 supplemented with 1% Ultraglutamine I, 1% Penicillin/Streptomycin, and 1.5 mg/ml Collagenase II.
FACS buffer	PBS supplemented with 1% EDTA (0.5 M) and 5 mg BSA.
Freezing medium	FBS with 10% DMSO
Washing buffer 1<br>For > 85% cell viability	PBS + 0.04% BSA
Washing buffer 2<br>For 70-85% cell viability	PBS + 1% BSA
Washing buffer 3<br>For < 70% cell viability	PBS + 10 FBS

Table S2 provided as an excel table and includes both high and low hierarchy cell type markers used to define immune cell types.

REFERENCES AND NOTES

1. S. L. Topalian, C. G. Drake, D. M. Pardoll, Immune checkpoint blockade: A common denominator approach to cancer therapy, *Cancer Cell* **27**, 450–461 (2015).

2. D. B. Doroshow, S. Bhalla, M. B. Beasley, L. M. Sholl, K. M. Kerr, S. Gnjatic, I. I. Wistuba, D. L. Rimm, M. S. Tsao, F. R. Hirsch, PD-L1 as a biomarker of response to immune-checkpoint inhibitors, *Nat. Rev. Clin. Oncol.* **18**, 345–362 (2021).

3. S. M. Toor, V. Sasidharan Nair, J. Decock, E. Elkord, Immune checkpoints in the tumor microenvironment, *Semin. Cancer Biol.* **65**, 1–12 (2020).

4. X. Zhou, S. C. Sun, Targeting ubiquitin signaling for cancer immunotherapy, *Signal Transduct. Target. Ther.* **6**, 16 (2021).

5. S. Qin, L. Xu, M. Yi, S. Yu, K. Wu, S. Luo, Novel immune checkpoint targets: Moving beyond PD-1 and CTLA-4, *Mol. Cancer* **18**, 155 (2019).

6. A. Rotte, J. Y. Jin, V. Lemaire, Mechanistic overview of immune checkpoints to support the rational design of their combinations in cancer immunotherapy, *Ann. Oncol.* **29**, 71–83 (2018).

7. R. W. Jenkins, A. R. Aref, P. H. Lizotte, E. Ivanova, S. Stinson, C. W. Zhou, M. Bowden, J. Deng, H. Liu, D. Miao, M. X. He, W. Walker, G. Zhang, T. Tian, C. Cheng, Z. Wei, S. Palakurthi, M. Bittinger, H. Vitzthum, J. W. Kim, A. Merlino, M. Quinn, C. Venkataramani, J. A. Kaplan, A. Portell, P. C. Gokhale, B. Phillips, A. Smart, A. Rotem, R. E. Jones, L. Keogh, M. Anguiano, L. Stapleton, Z. Jia, M. Barzily-Rokni, I. Canadas, T. C. Thai, M. R. Hammond, R. Vlahos, E. S. Wang, H. Zhang, S. Li, G. J. Hanna, W. Huang, M. P. Hoang, A. Piris, J. P. Eliane, A. O. Stemmer-Rachamimov, L. Cameron, M. J. Su, P. Shah, B. Izar, M. Thakuria, N. R. LeBoeuf, G. Rabinowits, V. Gunda, S. Parangi, J. M. Cleary, B. C. Miller, S. Kitajima, R. Thummalapalli, B. Miao, T. U. Barbie, V. Sivathanu, J. Wong, W. G. Richards, R. Bueno, C. H. Yoon, J. Miret, M. Herlyn, L. A. Garraway, E. M. Van Allen, G. J. Freeman, P. T. Kirschmeier, J. H. Lorch, P. A. Ott, F. S. Hodi, K. T. Flaherty, R. D. Kamm, G. M. Boland, K. K. Wong, D. Dornan, C. P. Paweletz, D. A. Barbie, Ex vivo profiling of PD-1 blockade using organotypic tumor spheroids, *Cancer Discov.* **8**, 196–215 (2018).

8. A. Aung, V. Kumar, J. Theprungsirikul, S. K. Davey, S. Varghese, An engineered tumor-on-a-chip device with breast cancer-immune cell interactions for assessing T-cell recruitment, *Cancer Res.* **80**, 263–275 (2020).

9. J. M. Ayuso, S. Rehman, M. Virumbrales-Munoz, P. H. McMinn, P. Geiger, C. Fitzgerald, T. Heaster, M. C. Skala, D. J. Beebe, Microfluidic tumor-on-a-chip model to evaluate the role of tumor environmental stress on NK cell exhaustion, *Sci. Adv.* **7**, eabc2331 (2021).

10. J. M. Ayuso, R. Truttschel, M. M. Gong, M. Humayun, M. Virumbrales-Munoz, R. Vitek, M. Felder, S. D. Gillies, P. Sondel, K. B. Wisinski, M. Patankar, D. J. Beebe, M. C. Skala, Evaluating natural killer cell cytotoxicity against solid tumors using a microfluidic model, *Onco Targets Ther* **8**, 1553477, (2019).

11. L. de Haan, J. Suijker, R. van Roey, N. Berges, E. Petrova, K. Queiroz, W. Strijker, T. Olivier, O. Poeschke, S. Garg, L. J. van den Broek, "A microfluidic 3D endothelium-on-a-chip model to study transendothelial migration of T cells in health and disease, *Int. J. Mol. Sci.* **22**, (2021).

12. M. Nguyen, A. De Ninno, A. Mencattini, F. Mermet-Meillon, G. Fornabaio, S. S. Evans, M. Cossutta, Y. Khira, W. Han, P. Sirven, F. Pelon, D. Di Giuseppe, F. R. Bertani, A. Gerardino, A. Yamada, S. Descroix, V. Soumelis, F. Mechta-Grigoriou, G. Zalcman, J. Camonis, E. Martinelli, L. Businaro, M. C. Parrini, Dissecting effects of anti-cancer drugs and cancer-associated fibroblasts by on-chip reconstitution of immunocompetent tumor microenvironments, *Cell Rep.* **25**, 3884–3893.e83, (2018).

13. C. M. Cattaneo, K. K. Dijkstra, L. F. Fanchi, S. Kelderman, S. Kaing, N. van Rooij, S. van den Brink, T. N. Schumacher, E. E. Voest, Tumor organoid-T-cell coculture systems, *Nat. Protoc.* **15**, 15–39, (2020).

14. S. Mensurado, R. Blanco-Dominguez, B. Silva-Santos, The emerging roles of gammadelta T cells in cancer immunotherapy, *Nat. Rev. Clin. Oncol.* **20**, 178–191 (2023).

15. D. H. Peng, B. L. Rodriguez, L. Diao, L. Chen, J. Wang, L. A. Byers, Y. Wei, H. A. Chapman, M. Yamauchi, C. Behrens, G. Raso, L. M. S. Soto, E. R. P. Cuentes, I. I. Wistuba, J. M. Kurie,

D. L. Gibbons, Collagen promotes anti-PD-1/PD-L1 resistance in cancer through LAIR1-dependent CD8(+) T cell exhaustion, *Nat. Commun.* **11**, 4520 (2020).

16. N. Jones, E. E. Vincent, J. G. Cronin, S. Panetti, M. Chambers, S. R. Holm, S. E. Owens, N. J. Francis, D. K. Finlay, C. A. Thornton, Akt and STAT5 mediate naive human CD4+ T-cell early metabolic response to TCR stimulation, *Nat. Commun.* **10**, 2042 (2019).

17. E. Bandala-Sanchez, Y. Zhang, S. Reinwald, J. A. Dromey, B. H. Lee, J. Qian, R. M. Bohmer, L. C. Harrison, T cell regulation mediated by interaction of soluble CD52 with the inhibitory receptor Siglec-10, *Nat. Immunol.* **14**, 741–748 (2013).

18. T. Yamauchi, T. Hoki, T. Oba, V. Jain, H. Chen, K. Attwood, S. Battaglia, S. George, G. Chatta, I. Puzanov, C. Morrison, K. Odunsi, B. H. Segal, G. K. Dy, M. S. Ernstoff, F. Ito, T-cell CX3CR1 expression as a dynamic blood-based biomarker of response to immune checkpoint inhibitors, *Nat. Commun.* **12**, 1402 (2021).

19. L. Sikkema, C. Ramirez-Suastegui, D. C. Strobl, T. E. Gillett, L. Zappia, E. Madissoon, N. S. Markov, L. E. Zaragosi, Y. Ji, M. Ansari, M. J. Arguel, L. Apperloo, M. Banchero, C. Becavin, M. Berg, E. Chichelnitskiy, M. I. Chung, A. Collin, A. C. A. Gay, J. Gote-Schniering, B. H. Kashani, K. Inecik, M. Jain, T. S. Kapellos, T. M. Kole, S. Leroy, C. H. Mayr, A. J. Oliver, M. von Papen, L. Peter, C. J. Taylor, T. Walzthoeni, C. Xu, L. T. Bui, C. De Donno, L. Dony, A. Faiz, M. Guo, A. J. Gutierrez, L. Heumos, N. Huang, I. L. Ibarra, N. D. Jackson, P. K. L. Murthy, M. Lotfollahi, T. Tabib, C. Talavera-Lopez, K. J. Travaglini, A. Wilbrey-Clark, K. B. Worlock, M. Yoshida, M. van den Berge, Y. Bosse, T. J. Desai, O. Eickelberg, N. Kaminski, M. A. Krasnow, R. Lafyatis, M. Z. Nikolic, J. E. Powell, J. Rajagopal, M. Rojas, O. Rozenblatt-Rosen, M. A. Seibold, D. Sheppard, D. P. Shepherd, D. D. Sin, W. Timens, A. M. Tsankov, J. Whitsett, Y. Xu, N. E. Banovich, P. Barbry, T. E. Duong, C. S. Falk, K. B. Meyer, J. A. Kropski, D. Pe'er, H. B. Schiller, P. R. Tata, J. L. Schultze, S. A. Teichmann, A. V. Misharin, M. C. Nawijn, M. D. Luecken, F. J. Theis, An integrated cell atlas of the lung in health and disease, *Nat. Med.* **29**, 1563–1577 (2023).

20. G. Middleton, K. Brock, J. Savage, R. Mant, Y. Summers, J. Connibear, R. Shah, C. Ottensmeier, P. Shaw, S. M. Lee, S. Popat, C. Barrie, G. Barone, L. Billingham, Pembrolizumab

in patients with non-small-cell lung cancer of performance status 2 (PePS2): A single arm, phase 2 trial, *Lancet Respir. Med.* **8**, 895–904 (2020).

21. M. Reck, D. Rodriguez-Abreu, A. G. Robinson, R. Hui, T. Csoszi, A. Fulop, M. Gottfried, N. Peled, A. Tafreshi, S. Cuffe, M. O'Brien, S. Rao, K. Hotta, K. Vandormael, A. Riccio, J. Yang, M. C. Pietanza, J. R. Brahmer, Updated analysis of KEYNOTE-024: Pembrolizumab versus platinum-based chemotherapy for advanced non-small-cell lung cancer with PD-L1 tumor proportion score of 50% or greater, *J. Clin. Oncol.* **37**, 537–546 (2019).

22. M. M. Awad, S. M. Gadgeel, H. Borghaei, A. Patnaik, J. C. Yang, S. F. Powell, R. D. Gentzler, R. G. Martins, J. P. Stevenson, M. Altan, S. I. Jalal, A. Panwalkar, M. Gubens, L. V. Sequist, S. Saraf, B. Zhao, B. Piperdi, and C. J. Langer, Long-term overall survival from KEYNOTE-021 cohort G: Pemetrexed and carboplatin with or without pembrolizumab as first-line therapy for advanced nonsquamous NSCLC, *J. Thorac. Oncol.* **16**, 162–168 (2021).

23. L. Gandhi, D. Rodriguez-Abreu, S. Gadgeel, E. Esteban, E. Felip, F. De Angelis, M. Domine, P. Clingan, M. J. Hochmair, S. F. Powell, S. Y. Cheng, H. G. Bischoff, N. Peled, F. Grossi, R. R. Jennens, M. Reck, R. Hui, E. B. Garon, M. Boyer, B. Rubio-Viqueira, S. Novello, T. Kurata, J. E. Gray, J. Vida, Z. Wei, J. Yang, H. Raftopoulos, M. C. Pietanza, M. C. Garassino, K. Investigators, Pembrolizumab plus chemotherapy in metastatic non-small-cell lung cancer, *N. Engl. J. Med.* **378**, 2078–2092 (2018).

24. R. Tang, W. Y. Langdon, J. Zhang, Regulation of immune responses by E3 ubiquitin ligase Cbl-b, *Cell. Immunol.* **340**, 103878 (2019).

25. R. C. Augustin, R. Bao, J. J. Luke, Targeting Cbl-b in cancer immunotherapy, *J. Immunother. Cancer* **11**, e006007 (2023).

26. J. M. Pitt, A. Marabelle, A. Eggermont, J. C. Soria, G. Kroemer, L. Zitvogel, Targeting the tumor microenvironment: Removing obstruction to anticancer immune responses and immunotherapy, *Ann. Oncol.* **27**, 1482–1492 (2016).

27. Y. Simoni, E. Becht, M. Fehlings, C. Y. Loh, S. L. Koo, K. W. W. Teng, J. P. S. Yeong, R. Nahar, T. Zhang, H. Kared, K. Duan, N. Ang, M. Poidinger, Y. Y. Lee, A. Larbi, A. J. Khng, E. Tan, C. Fu, R. Mathew, M. Teo, W. T. Lim, C. K. Toh, B. H. Ong, T. Koh, A. M. Hillmer, A. Takano, T. K. H. Lim, E. H. Tan, W. Zhai, D. S. W. Tan, I. B. Tan, E. W. Newell, Bystander CD8(+) T cells are abundant and phenotypically distinct in human tumour infiltrates, *Nature* **557**, 575–579 (2018).

28. K. K. Dijkstra, C. M. Cattaneo, F. Weeber, M. Chalabi, J. van de Haar, L. F. Fanchi, M. Slagter, D. L. van der Velden, S. Kaing, S. Kelderman, N. van Rooij, M. E. van Leerdam, A. Depla, E. F. Smit, K. J. Hartemink, R. de Groot, M. C. Wolkers, N. Sachs, P. Snaebjornsson, K. Monkhorst, J. Haanen, H. Clevers, T. N. Schumacher, E. E. Voest, Generation of tumor-reactive T cells by co-culture of peripheral blood lymphocytes and tumor organoids, *Cell* **174**, 1586–1598.e12 (2018).

29. K. Bachmaier, C. Krawczyk, I. Kozieradzki, Y. Y. Kong, T. Sasaki, A. Oliveira-dos-Santos, S. Mariathasan, D. Bouchard, A. Wakeham, A. Itie, J. Le, P. S. Ohashi, I. Sarosi, H. Nishina, S. Lipkowitz, J. M. Penninger, Negative regulation of lymphocyte activation and autoimmunity by the molecular adaptor Cbl-b, *Nature* **403**, 211–216 (2000).

30. Y. J. Chiang, H. K. Kole, K. Brown, M. Naramura, S. Fukuhara, R. J. Hu, I. K. Jang, J. S. Gutkind, E. Shevach, H. Gu, Cbl-b regulates the CD28 dependence of T-cell activation, *Nature* **403**, 216–220 (2000).

31. S. Han, Z. Q. Liu, D. C. Chung, M. S. Paul, C. R. Garcia-Batres, A. Sayad, A. R. Elford, M. J. Gold, N. Grimshaw, P. S. Ohashi, Overproduction of IFNgamma by Cbl-b-deficient CD8+ T cells provides resistance against regulatory T cells and induces potent antitumor immunity, *Cancer Immunol. Res.* **10**, 437–452 (2022).

32. X. Hu, J. Wang, M. Chu, Y. Liu, Z. W. Wang, X. Zhu, Emerging role of ubiquitination in the regulation of PD-1/PD-L1 in cancer immunotherapy, *Mol. Ther.* **29**, 908–919 (2021).

33. G. X. Zheng, J. M. Terry, P. Belgrader, P. Ryvkin, Z. W. Bent, R. Wilson, S. B. Ziraldo, T. D. Wheeler, G. P. McDermott, J. Zhu, M. T. Gregory, J. Shuga, L. Montesclaros, J. G. Underwood,

D. A. Masquelier, S. Y. Nishimura, M. Schnall-Levin, P. W. Wyatt, C. M. Hindson, R. Bharadwaj, A. Wong, K. D. Ness, L. W. Beppu, H. J. Deeg, C. McFarland, K. R. Loeb, W. J. Valente, N. G. Ericson, E. A. Stevens, J. P. Radich, T. S. Mikkelsen, B. J. Hindson, J. H. Bielas, Massively parallel digital transcriptional profiling of single cells, *Nat. Commun.* **8**, 14049 (2017).

34. Y. Hao, T. Stuart, M. H. Kowalski, S. Choudhary, P. Hoffman, A. Hartman, A. Srivastava, G. Molla, S. Madad, C. Fernandez-Granda, R. Satija, Dictionary learning for integrative, multimodal and scalable single-cell analysis, *Nat. Biotechnol.* **42**, 293–304 (2023).

35. M. Ester, H.-P. Kriegel, J. Sander, X. Xu, A density-based algorithm for discovering clusters in large spatial databases with noise, in *The Proceedings of the Second International Conference on Knowledge Discovery and Data Mining*, (AAAI Press, 1996).

36. L. Van der Maaten, G. Hinton, Visualizing data using t-SNE, *J. Mach. Learn. Res.* **9**, 2579–2605 (2008).

37. L. McInnes, J. Healy, N. Saul, L. Großberger, UMAP: Uniform Manifold Approximation and Projection, *J. Open Source Softw.* **3**, 861 (2018).

38. L. Waltman, N. J. van Eck, A smart local moving algorithn for large-scale modularity-based community detection, *Eur. Phys. J. B* **86**, 40829 (2013).

39. E. Cano-Gamez, B. Soskic, T. I. Roumeliotis, E. So, D. J. Smyth, M. Baldrighi, D. Wille, N. Nakic, J. Esparza-Gordillo, C. G. C. Larminie, P. G. Bronson, D. F. Tough, W. C. Rowan, J. S. Choudhary, G. Trynka, Single-cell transcriptomics identifies an effectorness gradient shaping the response of CD4(+) T cells to cytokines, *Nat. Commun.* **11**, 1801 (2020).

40. Y. Chu, E. Dai, Y. Li, G. Han, G. Pei, D. R. Ingram, K. Thakkar, J. J. Qin, M. Dang, X. Le, C. Hu, Q. Deng, A. Sinjab, P. Gupta, R. Wang, D. Hao, F. Peng, X. Yan, Y. Liu, S. Song, S. Zhang, J. V. Heymach, A. Reuben, Y. Y. Elamin, M. P. Pizzi, Y. Lu, R. Lazcano, J. Hu, M. Li, M. Curran, A. Futreal, A. Maitra, A. A. Jazaeri, J. A. Ajani, C. Swanton, X. D. Cheng, H. A. Abbas, M. Gillison, K. Bhat, A. J. Lazar, M. Green, K. Litchfield, H. Kadara, C. Yee, L. Wang, Pan-

cancer T cell atlas links a cellular stress response state to immunotherapy resistance, *Nat. Med.* **29**, 1550–1562 (2023).

41. P. L. Germain, A. Lun, C. Garcia Meixide, W. Macnair, M. D. Robinson, Doublet identification in single-cell sequencing data using scDblFinder, *F1000Res* **10**, 979 (2021).

42. Y. Hao, S. Hao, E. Andersen-Nissen, W. M. Mauck, 3rd, S. Zheng, A. Butler, M. J. Lee, A. J. Wilk, C. Darby, M. Zager, P. Hoffman, M. Stoeckius, E. Papalexi, E. P. Mimitou, J. Jain, A. Srivastava, T. Stuart, L. M. Fleming, B. Yeung, A. J. Rogers, J. M. McElrath, C. A. Blish, R. Gottardo, P. Smibert, R. Satija, Integrated analysis of multimodal single-cell data, *Cell* **184**, 3573–3587.e29 (2021).

43. C. D. Conde, C. Xu, L. B. Jarvis, D. B. Rainbow, S. B. Wells, T. Gomes, S. K. Howlett, O. Suchanek, K. Polanski, H. W. King, L. Mamanova, N. Huang, P. A. Szabo, L. Richardson, L. Bolt, E. S. Fasouli, K. T. Mahbubani, M. Prete, L. Tuck, N. Richoz, Z. K. Tuong, L. Campos, H. S. Mousa, E. J. Needham, S. Pritchard, T. Li, R. Elmentaite, J. Park, E. Rahmani, D. Chen, D. K. Menon, O. A. Bayraktar, L. K. James, K. B. Meyer, N. Yosef, M. R. Clatworthy, P. A. Sims, D. L. Farber, K. Saeb-Parsy, J. L. Jones, S. A. Teichmann, Cross-tissue immune cell analysis reveals tissue-specific features in humans, *Science* **376**, eabl5197 (2022).

44. D. Aran, A. P. Looney, L. Liu, E. Wu, V. Fong, A. Hsu, S. Chak, R. P. Naikawadi, P. J. Wolters, A. R. Abate, A. J. Butte, M. Bhattacharya, Reference-based analysis of lung single-cell sequencing reveals a transitional profibrotic macrophage, *Nat. Immunol.* **20**, 163–172 (2019).

45. N. A. Mabbott, J. K. Baillie, H. Brown, T. C. Freeman, D. A. Hume, An expression atlas of human primary cells: Inference of gene function from coexpression networks, *BMC Genomics* **14**, 632 (2013).

46. E. P. Consortium, An integrated encyclopedia of DNA elements in the human genome, *Nature* **489**, 57–74 (2012).

47. J. H. Martens, H. G. Stunnenberg, BLUEPRINT: Mapping human blood cell epigenomes, *Haematologica* **98**, 1487–1489 (2013).

48. J. Huuhtanen, D. Bhattacharya, T. Lonnberg, M. Kankainen, C. Kerr, J. Theodoropoulos, H. Rajala, C. Gurnari, T. Kasanen, T. Braun, A. Teramo, R. Zambello, M. Herling, F. Ishida, T. Kawakami, M. Salmi, T. Loughran, J. P. Maciejewski, H. Lahdesmaki, T. Kelkka, S. Mustjoki, Single-cell characterization of leukemic and non-leukemic immune repertoires in CD8(+) T-cell large granular lymphocytic leukemia, *Nat. Commun.* **13**, 1981 (2022).

49. R. Lopez, J. Regier, M. B. Cole, M. I. Jordan, N. Yosef, Deep generative modeling for single-cell transcriptomics, *Nat. Methods* **15**, 1053–1058 (2018).

50. I. Korsunsky, N. Millard, J. Fan, K. Slowikowski, F. Zhang, K. Wei, Y. Baglaenko, M. Brenner, P. R. Loh, S. Raychaudhuri, Fast, sensitive and accurate integration of single-cell data with Harmony, *Nat. Methods* **16**, 1289–1296 (2019).

51. N. L. de Vries, J. van de Haar, V. Veninga, M. Chalabi, M. E. Ijsselsteijn, M. van der Ploeg, J. van den Bulk, D. Ruano, J. G. van den Berg, J. B. Haanen, L. J. Zeverijn, B. S. Geurts, G. F. de Wit, T. W. Battaglia, H. Gelderblom, H. M. W. Verheul, T. N. Schumacher, L. F. A. Wessels, F. Koning, N. de Miranda, E. E. Voest, gammadelta T cells are effectors of immunotherapy in cancers with HLA class I defects, *Nature* **613**, 743–750 (2023).

52. F. Halary, M. A. Peyrat, E. Champagne, M. Lopez-Botet, A. Moretta, L. Moretta, H. Vie, J. J. Fournie, M. Bonneville, Control of self-reactive cytotoxic T lymphocytes expressing gamma delta T cell receptors by natural killer inhibitory receptors, *Eur. J. Immunol.* **27**, 2812–2821 (1997).

53. Y. Benjamini, Y. Hochberg, Controlling the false discovery rate: A practical and powerful approach to multiple testing, *J. R. Stat. Soc. B. Methodol.* **57**, 289–300 (1995).

54. M. Lotfollahi, M. Naghipourfar, M. D. Luecken, M. Khajavi, M. Buttner, M. Wagenstetter, Z. Avsec, A. Gayoso, N. Yosef, M. Interlandi, S. Rybakov, A. V. Misharin, F. J. Theis, Mapping single-cell data to reference atlases by transfer learning, *Nat. Biotechnol.* **40**, 121–130 (2022).

55. I. Tirosh, B. Izar, S. M. Prakadan, M. H. Wadsworth, 2nd, D. Treacy, J. J. Trombetta, A. Rotem, C. Rodman, C. Lian, G. Murphy, M. Fallahi-Sichani, K. Dutton-Regester, J. R. Lin, O. Cohen, P.

Shah, D. Lu, A. S. Genshaft, T. K. Hughes, C. G. Ziegler, S. W. Kazer, A. Gaillard, K. E. Kolb, A. C. Villani, C. M. Johannessen, A. Y. Andreev, E. M. Van Allen, M. Bertagnolli, P. K. Sorger, R. J. Sullivan, K. T. Flaherty, D. T. Frederick, J. Jane-Valbuena, C. H. Yoon, O. Rozenblatt-Rosen, A. K. Shalek, A. Regev, L. A. Garraway, Dissecting the multicellular ecosystem of metastatic melanoma by single-cell RNA-seq, *Science* **352**, 189–196 (2016).

56. N. Borcherding, N. L. Bormann, G. Kraus, scRepertoire: An R-based toolkit for single-cell immune receptor analysis, *F1000Res* **9**, 47 (2020).

57. Y. Zhou, B. Zhou, L. Pache, M. Chang, A. H. Khodabakhshi, O. Tanaseichuk, C. Benner, S. K. Chanda, Metascape provides a biologist-oriented resource for the analysis of systems-level datasets, *Nat. Commun.* **10**, 1523 (2019).

58. M. Kanehisa, M. Furumichi, Y. Sato, M. Kawashima, M. Ishiguro-Watanabe, KEGG for taxonomy-based analysis of pathways and genomes, *Nucleic Acids Res.* **51**, D587-D592 (2023).

59. M. Milacic, D. Beavers, P. Conley, C. Gong, M. Gillespie, J. Griss, R. Haw, B. Jassal, L. Matthews, B. May, R. Petryszak, E. Ragueneau, K. Rothfels, C. Sevilla, V. Shamovsky, R. Stephan, K. Tiwari, T. Varusai, J. Weiser, A. Wright, G. Wu, L. Stein, H. Hermjakob, P. D'Eustachio, The Reactome Pathway Knowledgebase 2024, *Nucleic Acids Res.* **52**, D672–D678 (2024).

60. M. A. DePristo, E. Banks, R. Poplin, K. V. Garimella, J. R. Maguire, C. Hartl, A. A. Philippakis, G. del Angel, M. A. Rivas, M. Hanna, A. McKenna, T. J. Fennell, A. M. Kernytsky, A. Y. Sivachenko, K. Cibulskis, S. B. Gabriel, D. Altshuler, M. J. Daly, A framework for variation discovery and genotyping using next-generation DNA sequencing data, *Nat. Genet.* **43**, 491–498 (2011).

61. K. J. Karczewski, L. C. Francioli, G. Tiao, B. B. Cummings, J. Alfoldi, Q. Wang, R. L. Collins, K. M. Laricchia, A. Ganna, D. P. Birnbaum, L. D. Gauthier, H. Brand, M. Solomonson, N. A. Watts, D. Rhodes, M. Singer-Berk, E. M. England, E. G. Seaby, J. A. Kosmicki, R. K. Walters, K. Tashman, Y. Farjoun, E. Banks, T. Poterba, A. Wang, C. Seed, N. Whiffin, J. X. Chong, K. E. Samocha, E. Pierce-Hoffman, Z. Zappala, A. H. O'Donnell-Luria, E. V. Minikel, B. Weisburd,

M. Lek, J. S. Ware, C. Vittal, I. M. Armean, L. Bergelson, K. Cibulskis, K. M. Connolly, M. Covarrubias, S. Donnelly, S. Ferriera, S. Gabriel, J. Gentry, N. Gupta, T. Jeandet, D. Kaplan, C. Llanwarne, R. Munshi, S. Novod, N. Petrillo, D. Roazen, V. Ruano-Rubio, A. Saltzman, M. Schleicher, J. Soto, K. Tibbetts, C. Tolonen, G. Wade, M. E. Talkowski, B. M. Neale, M. J. Daly, D. G. MacArthur, C. Genome Aggregation Database, The mutational constraint spectrum quantified from variation in 141,456 humans, *Nature* **581**, 434–443 (2020).

62. K. Wang, M. Li, H. Hakonarson, ANNOVAR: Functional annotation of genetic variants from high-throughput sequencing data, *Nucleic Acids Res.* **38**, e164 (2010).

63. J. G. Tate, S. Bamford, H. C. Jubb, Z. Sondka, D. M. Beare, N. Bindal, H. Boutselakis, C. G. Cole, C. Creatore, E. Dawson, P. Fish, B. Harsha, C. Hathaway, S. C. Jupe, C. Y. Kok, K. Noble, L. Ponting, C. C. Ramshaw, C. E. Rye, H. E. Speedy, R. Stefancsik, S. L. Thompson, S. Wang, S. Ward, P. J. Campbell, S. A. Forbes, COSMIC: The catalogue of somatic mutations in cancer, *Nucleic Acids Res.* **47**, D941-D947 (2019).

64. J. Cheng, G. Novati, J. Pan, C. Bycroft, A. Zemgulyte, T. Applebaum, A. Pritzel, L. H. Wong, M. Zielinski, T. Sargeant, R. G. Schneider, A. W. Senior, J. Jumper, D. Hassabis, P. Kohli, Z. Avsec, Accurate proteome-wide missense variant effect prediction with AlphaMissense, *Science* **381**, eadg7492 (2023).

65. D. Sha, Z. Jin, J. Budczies, K. Kluck, A. Stenzinger, F. A. Sinicrope, Tumor mutational burden as a predictive biomarker in solid tumors, *Cancer Discov.* **10**, 1808–1825 (2020).
